# Longitudinal Distribution Map of the Active Components and Endophytic Fungi in *Angelica sinensis* (Oliv.) Diels Root and Their Potential Correlations

**DOI:** 10.3390/metabo14010048

**Published:** 2024-01-12

**Authors:** Ying Sun, Rong Guo, Yuting Geng, Hushan Shang, Xiaopeng Guo, Yue Wu, Yonggang Wang, Li Li, Xuee Li, Shengli Zhang, Ning Xu, Xueyan Li

**Affiliations:** 1 Gansu Institute for Drug Control, Lanzhou 730070, China; 2 School of Life Science and Engineering, Lanzhou University of Technology, Lanzhou 730050, China; 3 Department of Water Resources of Gansu Province, Lanzhou 730020, China; 4 Dingxi Academy of Agricultural Sciences, Dingxi 743002, China

**Keywords:** Chinese Angelica, different medicinal parts, endophytic fungal communities, active metabolic components, spatial variations, association analysis

## Abstract

The three distinct medicinal parts of *Angelica sinensis* (Oliv.) Diels (Ang) roots are the head, body, and tail (ARH, ARB, and ART, respectively). How endophytic fungi shape the differences in metabolic components among these parts remains unclear. We quantified the distribution of active components and endophytic fungi along the ARH, ARB, and ART and their relationships. Based on the metabolic components and their abundances detected via non-target metabolism, the different medicinal parts were distinguishable. The largest number of dominant metabolic components was present in ART. The difference between ART and ARH was the greatest, and ARB was in a transitional state. The dominant active molecules in ART highlight their effects in haemodynamics improvement, antibacterial, anti-inflammatory, and hormone regulation, while ARH and ARB indicated more haemostasis, blood enrichment, neuromodulation, neuroprotection and tranquilisation, hepatoprotection, and antitumour activities than that of ART. The ARHs, ARBs, and ARTs can also be distinguished from each other based on the endophytic fungi at the microbiome level. The most dominant endophytic fungi were distributed in ART; the differences between ART and ARH were the largest, and ARB was in a transition state, which is consistent with the metabolite distributions. Structural equation modelling showed that the endophytic fungi were highly indicative of the metabolic components. Correlation analysis further identified the endophytic fungi significantly positively correlated with important active components, including *Condenascus tortuosus*, *Sodiomyces alcalophilus*, and *Pleotrichocladium opacum*. The bidirectional multivariate interactions between endophytic fungi and the metabolic components shape their spatial variations along the longitudinal direction in the Ang root.

## 1. Introduction

**Figure 1:**
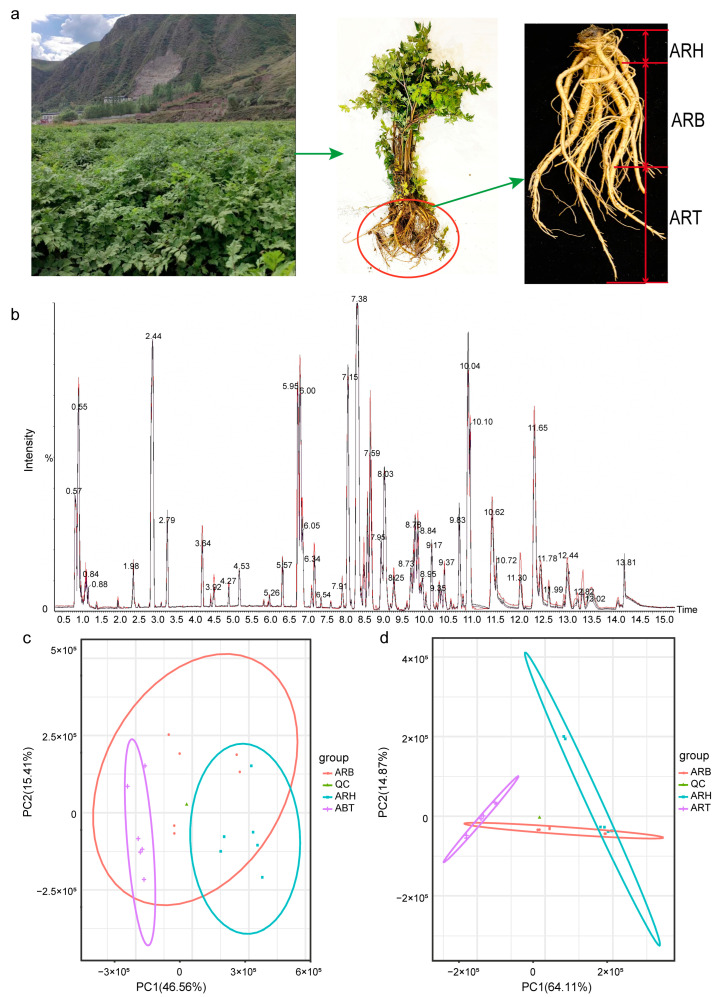
Sampling and system stability of the metabonomic determination for Ang root and the discrimination of different medicinal parts based on the results. (**a**) Morphological divisions in the different medicinal parts of Ang root indicate the sample collection areas for the different parts. (**b**) Peaks of all QC sample outputs determined using the LC-MS system (positive ion mode) almost overlap, indicating the stability of the system; c and d, PCA and clustering of samples from different medicinal parts based on metabolite abundance data obtained from the positive (**c**) and negative (**d**) ion models.

The dried root of *Angelica sinensis* (Oliv.) Diels (Ang), commonly known as Chinese Angelica in traditional Chinese medicine (TCM), has long been used to treat gynaecological diseases because it enriches the blood, promotes blood circulation, regulates menstruation, relieves pain, and relaxes the bowels. More importantly, Ang exhibits antitumour, antioxidant, bacteriostatic, and antiviral effects and thus has extensive medicinal value [1,2]. It is a commonly used drug in TCM prescriptions and modern clinics. High-output, good-quality, and authentic Ang is produced in Minxian County, Gansu Province, China [3]. The root, the source of the medication, is slightly cylindrical with a light brown to brown surface. The upper part of the Ang root (ARH) is called the root head, and it has a ring pattern, and the upper end is round and blunt; the taproot, also known as the Ang root body (ARB), has an uneven surface and branching roots in its lower part, called the Ang root tail (ART), which is thick at the top and thin at the bottom and has a few fibrous root marks [4] (Figure 1a).

The ARH, ARB, and ART have different medicinal effects. The TCM theory and clinical experience indicate that ARH, ARB, and ART aid in haemostasis, enriching blood, and promoting blood circulation, respectively; whole Ang root can be used to harmonise blood (i.e., enriching blood and promoting blood circulation) [5]. Modern pharmacological studies have confirmed this hypothesis. The active ingredients of Ang root that promote blood circulation include n-butenyl phthalide, ligustilide, and ferulic acid. Quantitative chemical analysis of different medicinal parts showed that the content of blood-activating components in ART increased significantly [6], and those in ARH decreased significantly. This is consistent with the results of modern pharmacological research showing that Ang root is divided into different parts for use as medicine. Details regarding the spatially specific distribution pattern of the active medicinal components in Ang root have been provided in the literature. For example, Li et al. [7] identified 36 types of primary and secondary metabolites in Ang roots and analysed the main components distributed in different parts. However, the chemical composition of Ang root is complex, and fully identifying the differences among ARH, ARB, and ART based on a few components is difficult.

Tissue-dependent differences in metabolic patterns and the efficacy of medicinal plants may also be due to various complicated factors. For example, in addition to genetic differentiation and selective expression, microecology is an important factor [8,9]. In recent years, the variations in the endophytes in the different parts of the plant have received considerable attention. For example, studies have shown large differences in the endophytic fungi in the different tissues of *Polygala fallax* Hemsl [10] and that there are more endophytic fungi in the roots of *Ferula* species than in the stems and leaves [11]. Related studies have also shown that different endophytes occupy different niches and that the community structures of the endophytic microbes can show large amounts of variation. The interactions between endophytes and medicinal plants have been an ongoing and hot topic area of research [12]. Endophytes play an important role in shaping the accumulation patterns of medicinally active ingredients in the host by promoting the secondary metabolism, inhibiting pathogenic microorganisms, functioning as a biocontrol, enhancing the resistance of the host to abiotic stressors, and producing new structural analogues of the host active ingredients through microbial transformations [13,14]. The active components of medicinal plants can affect the assembly of endophytic microbiota and shape the community structure [15]. For example, studies have revealed that triterpenoids from *Schisandra chinensis* regulate the composition of the endophytic community and promote plant growth and metabolite accumulation [16]. Notably, plants have successfully evolved means of survival, which has involved the secretion of byproducts and selecting endophytic microbiota that are beneficial to themselves as much as possible [17].

The tissue dependence on medicinal plant active ingredients and endophytes has been confirmed in several studies [18,19]. Therefore, we can speculate that the drug-active ingredients and microorganisms distinguished by different tissue parts may further shape their interrelated tissue specificity through various plant-microorganism interactions. Recent studies have revealed differences in some medicinally active components and endophytes in different tissue parts simultaneously and analysed the correlation between them. Chen et al. revealed differences in the richness and abundance of endophyte species in different tissues of *Gentiana straminea* Maxim, showing a significant positive correlation with gentiopicroside [20].

Although different medicinal parts of Ang root have distinct pharmacological characteristics, according to our review of the literature, no studies have investigated the spatial variations in endophytes and their potential association with the active components. Endophytic fungi have been widely revealed in medicinal plants, although the role of endophytic bacteria is equally important [10,12,13,14,21,22]. Furthermore, the active components of the different parts of Ang root must be supplemented at a metabolomic level. Multi-omics association analysis is a valuable tool for identifying key factors in microecology [23,24,25]. For example, the combination of the microbiome and metabolome helps identify key microorganisms and metabolites in microecosystems and their potential interrelated information [25].

Therefore, deepening the understanding of how Ang root shapes its endophytic microbiota based on different active components in various medicinal parts and how the spatially specific distribution of endophytes affects medicinally active ingredients and clarifying the key microecological factors are important. Thus, in this study, we combined microbiome and metabolome analyses and revealed the characteristics of the endophytic fungal community and metabolite composition patterns in different medicinal parts of the Ang root and their potential associations. This study contributes to the literature because it investigates different adjacent parts in the same tissue (Ang root) and obtains more comprehensive spatial dependence information regarding metabolites and microorganisms than that is available in the literature. Our findings provide a reference for modern applications of Ang, including precision medication, microecological quality control, discovery of the active ingredient, and microbial transformation.

## 2. Materials and Methods

### 2.1. Collection of Fresh Plants and Preparation of Samples

Study samples were collected from the Ang planting area located in Minxian County, Dingxi City, Gansu Province, from the core plot in an authentic production area. The variety of Ang samples collected is Mingui No. 1, which is provided by Gansu Minxian Danggui Research Institute (Dingxi City, China), and is also the main planting variety in Minxian County. The collection period was in October, the harvest period for Ang. First, the whole plant was dug out by the planting roots. Next, six Ang roots were randomly selected from those that appeared healthy; they had no disease, good growth, and relatively consistent form. These six Ang roots were placed in sterile plastic bags and quickly transported to the laboratory at a low temperature. Each root was then sampled as follows: the soil attached to the root was carefully shaken off; the root surface was rinsed with running water and then wiped with sterile paper; the root head, body, and tail regions were distinguished based on their morphology (Figure 1a); and ~100 g of tissue was cut from each of the corresponding segments.

To prepare the samples for metabolome detection, medicinal parts (i.e., ARH, ARB, and ART) were collected on an ultra-clean platform, cut into ~8 mm^3^ pieces, mixed, and packed separately with 1 g per tube for cryopreservation. For endophytic fungi determination, the longer tissues were cut in the corresponding regions, and the surfaces were washed and disinfected, as previously described [26,27]. The tissues for the different parts were chopped and mixed evenly and then packed and frozen, 1 g per tube.

### 2.2. Metabolite Extraction

Frozen samples (storage time ~15 days) from the parts of the Ang roots were ground, and the extract was added (methanol-acetonitrile volume ratio = 1:1, internal standard concentration 2 mg/L). After ultrasonic treatment, they were left to stand at −20 °C and centrifuged at 12,000 rpm (~13,400× *g*); next, the supernatant was collected. After drying the extract in a vacuum concentrator, 160 μL of solution (acetonitrile-water volume ratio: 1:1) was added for re-dissolution. After vortex mixing, ultrasonic treatment in the ice water bath, and centrifugation, 120 μL supernatant was collected and injected into a 2 mL injection bottle, and 10 μL of each sample was mixed into the quality control (QC) samples for detection.

### 2.3. Detection and Identification of Metabolites

The liquid chromatography–mass spectrometry (LC-MS) system for the determination of metabolomics consisted of PLUS ultra-performance liquid chromatography (UHPLC) (Acquity I-Class, Waters, Taunton, MA, USA) and high-resolution mass spectrometry (HRMS) (Xevo G2-XS QTOF, Waters, Taunton, MA, USA). The column used was an Acquity UPLC HSS T3 (1.8 um 2.1 × 100 mm) from Waters. In the positive and negative ion modes, 0.1% formic acid aqueous solution was used as mobile phase A, 0.1% acetonitrile formate was used as mobile phase B, and the injection volume was 1 μL. Primary and secondary mass spectrometry data were collected in the MSe mode under the control of acquisition software (MassLynx V4.2, Waters), and dual-channel data were simultaneously collected for low collision energy (2 V) and high collision energy (10–40 V). For each mass spectrometer, the scanning frequency was 0.2 s. Peak extraction and alignment were performed using Progenesis QI software (v3.0) with raw data collected by MassLynx v4.2. Peak identification was based on the online METLIN database attached to the Progenesis QI software (v3.0) and BioMarker’s self-built database [28]. In a mass spectrometry (MS) system, molecular cleavage patterns follow certain rules and contain valuable information about the chemical structures. Therefore, we used the MS-Fragmenter demo software (v2017.2) to predict compound ion fragments through MS fragmentation rules established in the literature [29]. This can clarify fragmentation pathways and fragment structures, and the structures can be confirmed by comparing the predicted MS fragments of compounds with the real experimental spectra.

In order to capture and label as many differential metabolic components as possible and preliminarily reveal their distribution patterns among various medicinal parts in Ang root on a whole level, we first set a relatively loose deviation in the ion mass number, that is, the deviation in the parent ion mass number was 100 ppm, while the deviation in the fragment ion mass number was <50 ppm. However, when determining the dominant metabolites in different medicinal parts, we filtered out the metabolites with a large deviation in the parent ion mass number by qualitative scores. The parent ion mass number deviation for the dominant metabolites identified in various medicinal sites does not exceed 15 ppm. After completing peak extraction and alignment via the Progenesis QI workflow and obtaining a fragment ion spectrogram using the MS-Fragmenter demo software (v2017.2), the compound ID was obtained based on the Metascope search engine built in Progenesis QI, and then compound information was comprehensively analysed, including parent and daughter ions, retention time, declustering potential, and collision energy. Combining these parameters, a qualitative score and corresponding qualitative grade were output for each compound, which then served as the basis for screening conservatively identified compounds. In order to ensure the reliability of the analytical results, we first highlighted the compounds known as phytochemical components with scores greater than 0.9. In addition, according to the identification information, some metabolites are considered structural analogues of synthetic drugs. Among them, we retained and labelled metabolites with high scores and large differences among various medicinal parts and assumed efficacy consistent with the representative functions of their main distribution parts, which ensured the rigour of the analysis results.

### 2.4. Metabonomic Analysis

The original peak areas for each metabolite were normalised using the Progenesis QI software (v3.0). This step was performed by dividing the metabolite of each sample by the total peak area for that sample and multiplying by the average of all the peak areas. The obtained values were used to characterise the expression intensities of the corresponding metabolites. Classification and pathway information for the identified metabolites were obtained using the Kyoto Encyclopedia of Genes and Genomes (KEGG), the human metabolome database (HMDB), and lipid maps databases [25]. Principal component analysis (PCA) was used to investigate the differences in metabolic patterns between and within groups and the repeatability of the QC samples. It was implemented using the ggord package and prcomp function of the R language tool (v4.0.5) (http://www.r-project.org/) (accessed on 9 September 2023). The orthogonal partial least squares-discriminant analysis (OPLS-DA)model was constructed using the R package ropls, and 200 permutation tests were performed to verify the model’s reliability. The VIP values of each metabolite in the model were calculated using multiple cross-validations. Based on the grouping information, the fold-changes (FCs) of the metabolites between different medicinal parts were calculated and compared. A *t*-test was used to calculate the significance (*p*-value) of the differences between the groups. Differential metabolites were screened by combining the FC, *p*, and VIP values. The screening criteria were FC > 2, *p* < 0.05, and VIP > 1. ClusterProfiler and hypergeometric tests were used to analyse the enrichment patterns of the KEGG terms with annotated differential metabolites. The enrichplot and ggplot2 R packages were used to draw an enrichment network diagram.

### 2.5. Metagenome Extraction, ITS Sequence Amplification, and Sequencing

The total DNA of the endophytic fungi in the different parts of the Ang roots was rapidly extracted using the E. Z. N. A. Fungal DNA Kit (OMEGA, Norcross, GA, USA) according to the manufacturer’s instructions. The purity, concentration, and integrity of the DNA were determined. After the DNA samples were tested, an amplicon sequencing of the fungal internal transcribed spacer (ITS) was performed. ITS1 (5′-CTTGGTCATTTAGAGGAAGTAA-3′) and ITS4 (5′-GCTGCGTTCTTCATGC-3′) were used as primers to amplify the ITS1 region of the fungi. The PCR amplification products were purified, quantified, and homogenised using the OMEGA Cycle Pure Kit (OMEGA, Norcross, GA, USA). According to the Illumina Novaseq Platform (Illumina, San Diego, CA, USA) standard operating procedure, the purified and amplified fragments were constructed into a small fragment library for paired-end sequencing, and the library was sequenced with an Illumina Novaseq 6000 after quality inspection. The original image data file was transformed into sequenced reads using a base-calling analysis, and the results were stored in a FASTQ file format. Data preprocessing was performed in three steps—quality filtering, double-ended sequence splicing, and chimaera removal—to obtain the final effective read length [30].

### 2.6. Microbial Diversity Analysis

USEARCH software was used to cluster the reads at a 97.0% similarity level, and the operational taxonomic units (OTUs) were obtained. With UNITE as the reference database, we used a naive Bayesian classifier to annotate the representative sequences, obtain the species classification information corresponding to each sequence, and count the fungal community compositions in each sample [31]. SPSS (version 26.0) was used for nonparametric tests to analyse the variance in the species abundance data of the different medicinal parts [32]. Based on the R language tool (v4.0.5) (http://www.r-project.org/) (accessed on 9 September 2023), the prcomp function was used to conduct PCA; the ggplot2 package was used to draw the PCA diagram; the Venn package was used to draw the Venn diagrams of different species between different medicinal tissues; and the pheatmap package was used to draw a clustered heatmap of the differential species among the ARH, ARB, and ART.

### 2.7. Correlation Analysis between the Microbiome and Metabolome

The mutual directivity of the first-level indicators (i.e., microbial community and metabolite composition patterns) and the directivity of the second-level indicators to the first-level indicators were investigated; the latter includes the directivity of different species to microbial modules and metabolites to metabolite modules. The methods used were as follows: first, based on the partial least squares method, path analysis and data visualisation were performed using the R packages plspm and ggplot2, and a structural equation model was constructed [33], and second, Pearson correlation analysis of differential microbial species and metabolites was performed using the cor.test function and corrplot package with the R language [34].

### 2.8. Data Analysis

Three repetitions were used for the microbiome assay; six repetitions were used for the metabolome assay. Regardless of the metabolic components or microbial species, the significance of the differences between the medicinal parts was evaluated using analysis of variance with IBM SPSS Statistics version 26.0, and *p* < 0.05 was considered significant.

## 3. Results

### 3.1. Metabolite Information for Ang Root and the Metabolic Patterns of the Different Medicinal Parts

A total of 10,618 peaks and 778 metabolites were detected in the UHPLC-HRMS-positive ion mode from 18 ARH, ARB, and ART samples and 6 QC samples. A total of 6409 peaks were detected in the negative ion mode, and 313 metabolites were annotated (Supplementary Data Table S1). The response intensity and retention time of the QC samples in the positive and negative ion modes showed high reproducibility, reflecting the stability of the analysis system and ensuring the reliability of the experimental data (Figure 1b, Supplementary Data Figure S1). According to the HMDB annotation results of the positive and negative ion patterns, the main superclasses of the metabolites were organoheterocyclic compounds, lipids and lipid-like molecules, organic acids and derivatives, benzenoids, phenylprotopanoids and polyketides, nucleosides, nucleotides, and analogues, and organic oxygen compounds (Supplementary Data Table S1).

According to the HMDB categories that were detected and annotated, a preliminary finding was that most metabolites were potential natural products with biological activities, structural analogues, precursors, intermediates, and derivatives of drug molecules, or important functional molecules in metabolic network nodes [35,36,37] (Supplementary Data Table S1).

The PCA of the samples from the ARH, ARB, ART, and QC, based on the distribution patterns of the metabolite content, showed that six of the QC samples were closely aggregated and almost difficult to distinguish, indicating the stability of the metabolomic determination; for the samples from different medicinal parts, although they did not show the same high levels of aggregation as the QC samples, those from the same parts were relatively aggregated and located in a specific distinguishable two-dimensional area (Figure 1c,d). Notably, the ARH and ART samples were highly separated, and the ARB samples showed crosstalk between the ARH and ART. These clustering characteristics were consistent with the positive and negative ion modes. This result was also in accordance with the spatial distributions of the ARH, ARB, and ART because the ARB was in a transition state.

Furthermore, the OPLS-DA model was used to evaluate the inter- and intragroup differences. Overall, the samples of the two different medicinal parts were distributed on the left and right sides of the to1 axis, which could be strictly distinguished (Figure S2). Although the samples in the same parts were separated along the to1 axis, the discrimination between groups was obviously greater than that within groups in terms of the distribution characteristics of the samples in two-dimensional coordinates. Furthermore, objective intragroup variation reflected the differences between the different plants in the same root part. In this study, samples of the same medicinal parts were obtained from different plants with similar growth and quality. Therefore, because of the exclusion of plant specificity, the differential metabolites obtained later better reflected the differences in different medicinal parts.

### 3.2. Differential Metabolites between Different Medicinal Parts

**Figure 2:**
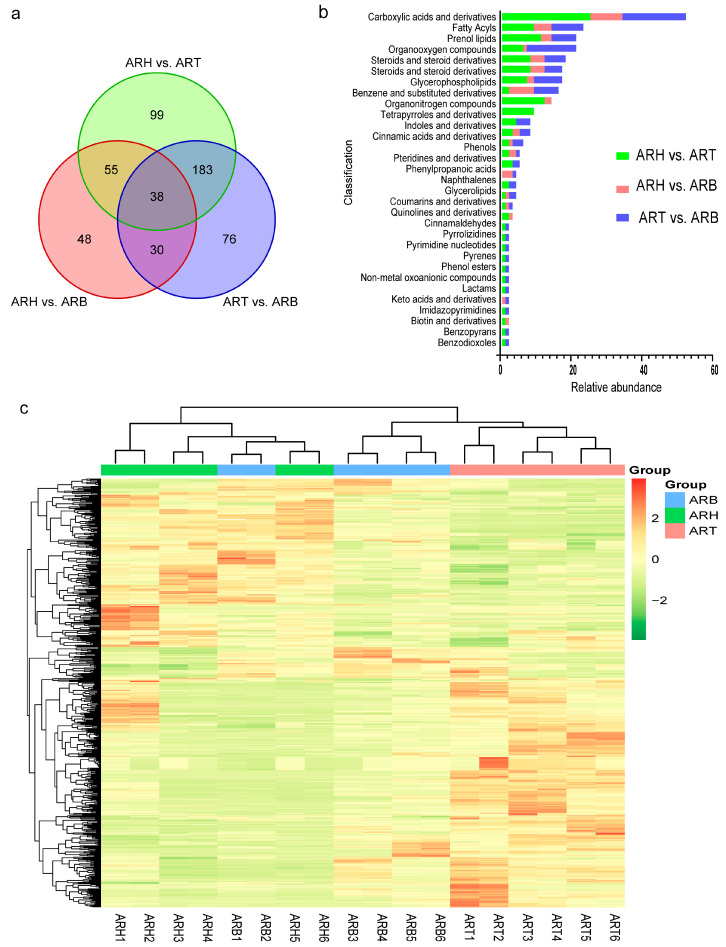
Analysis of the differential metabolites between pairs of medicinal parts in Ang root (ARH vs. ART, ARH vs. ARB, and ART vs. ARB) under a positive ion mode. (**a**) Venn diagram showing the differential metabolites obtained by pairwise comparison; (**b**) categorical distributions of the differential metabolites in the pairwise comparisons; (**c**) hierarchical clustering of samples from different medicinal parts based on the abundance data for the differential metabolites.

The metabolic components of the ARH, ARB, and ART were compared between the pairs. The number of differential metabolites (union) in the positive and negative ion modes was 529 and 204, respectively, which reached 67.99% and 65.17% of the identified metabolites (Figure 2a, Supplementary Data Figure S3a). Among these, the differential metabolites of ARH vs. ART were the highest, ARH vs. ARB were the lowest, and ARB vs. ART were intermediate. This variation law is in accordance with the spatial relationships among ARH, ARB, and ART, and ARB is in a transitional state. There were also differences in the types of differential metabolites between the pairs (Figure 2b, Supplementary Data Figure S3b). Regardless of their ion mode, the first five categories with the most differential metabolites showed different results for the three pairwise comparisons. Hierarchical clustering was performed for all samples based on the distributions of different metabolites in the different medicinal parts, and those from the same parts were gathered together. There was a large proportion of metabolites for which the content was the highest in the ART or ARH, especially in the former, and the number of metabolites with the highest content in ARB was the lowest (Figure 2c, Supplementary Data Figure S3c). The distribution patterns of the differential metabolites also showed that ARH and ART were relatively distant, and the ARB was in a transitional state.

The biological activities of the differential metabolites were annotated based on the literature and databases, including the Traditional Chinese Medicine Systems Pharmacology Database and Analysis Platform (TCMSP). Pairwise comparisons between the different medicinal parts highlighted some of the top differentially expressed metabolites. In the ARH and ART comparison, the log_2_FC of the top 10 differential metabolites in the positive ion mode ranged from 7 to 30, with an average of 20.04 (Table 1). Among the upregulated metabolites in ART, corchorusoside E, 6-pentadecyl salicylic acid, ononin, and hexadecyl ferulate are known phytochemical constituents and have been revealed to have blood-activating effects. Specifically, corchorusoside E belongs to cardiac glycosides, which can improve heart failure and promote cardiovascular microcirculation; 6-pentadecyl salicylic acid and ononin, as typical active ingredients in Chinese herbal medicine (CHM), not only can improve cardiovascular function but also have multiple effects such as antioxidant, antibacterial, anti-inflammatory, and antitumour effects; and hexadecyl ferulate is a derivative of ferulic acid, which may have the potential effects of inhibiting platelet aggregation, antithrombosis, anti-inflammation, and antioxidation. In addition, the pos_8029, characterised as a potential structural analogue of U-73122 with a higher score, may be a potential blood-activating component because U-73122 can reduce blood lipids and has anticancer effects [35,37].

Among the upregulated active ingredients in ARH, viburtinal has the effects of haemostasis and nerve regulation and protection and has anticancer and anti-inflammation effects; tryptamine has a haemostatic effect based on vasoconstriction; anethole can promote leukocyte proliferation and bone marrow cell maturation and release and is also related to the improvement of neuropsychiatric diseases (e.g., depression) [35,37]. In the negative ion mode, the Log_2_FC of the top 10 differential metabolites ranged from 2 to 24, with an average of 15.125 (Supplementary Data Table S2). Naringenin-7-O-glucoside and compactin (secondary metabolites of fungi in the medicinal plant-microbial ecosystem) are upregulated in ART and promote blood circulation. The former also regulates the menstrual cycle, resisting oxidation and tumour growth, and the latter has antifungal activity [37]. Among other upregulated metabolites in ART, procyanidin C1, a typical active ingredient in CHM, can resist ageing, improve blood circulation, reduce swelling and blood stasis, and inhibit varicose veins; sappanone A dimethyl ether can inhibit neuroinflammation and ageing; quinorose, a typical CHM ingredient, has anti-inflammatory, antitumour, antibacterial, and antiviral effects; Genkwanin is also a typical active ingredient in CHM, which can lower blood pressure, promote uterine contraction, and improve coagulation function after postpartum haemorrhage and has preventive and therapeutic effects on diabetes and Alzheimer’s disease; neg_1428, characterised as a potential structural analogue of zidovudine, has potential as an antiviral compound [35,37]. Among the upregulated active ingredients in ARH, oleandrin and salannin, typical CHM active ingredients, have antitumour and antiviral effects; L-citrulline and L-lysine monohydrochloride are important nutrients that have therapeutic effects; manumycin A is a microbial antibiotic with anticancer efficacy, attributed to the medicinal plant-microbial ecosystem; and glutathione is a typical antioxidant [35,36,37].

Comparisons between ARB and ART show that the log_2_FC of the top 10 differential metabolites in the positive ion mode ranged from 3 to 26, with an average of 13.99 (Table S3). Among the upregulated active components in ART, corchorusoside E and 6-pentadecyl salicylic acid improved haemodynamics through different modes of action; pos_7434, identified as a potential structural analogue of 20-hydroxy-PGF2a, may also be a potential metabolite with blood circulation function [35,37]. Arginine, dipeptide, and tripeptide, which are usually considered nutrients and physiological regulators [36], were upregulated in ARB. Furthermore, the tryptamine content in ARB was higher than that in ART and similar to that in ARH. In the negative ion mode, the Log_2_FC of the top 10 differential metabolites ranged from 3 to 24, with an average of 11.12 (Supplementary Data Table S3). Gentiobiose was the metabolite with the highest level of upregulation in ART. As a prebiotic, gentiobiose can promote the proliferation of *Bifidobacterium* and has various functions, such as reducing blood endotoxins, lowering cholesterol concentration, preventing arteriosclerosis and hypertension, resisting tumours, and enhancing immunity [38]. L-citrulline was the metabolite with the largest upregulation range in ARB. As an α-amino acid with health care function, L-citrulline can also improve immunity and relieve fatigue and stress [36].

The comparison of ARH and ARB showed that the log_2_FC of the top 10 differential metabolites in the positive ion mode ranged from 1 to 18, with an average of 6.84 (Supplementary Data Table S4). Among the significantly upregulated active components in ARH, pinolenic acid and flavanone are included in the TCMSP database and are related to the prevention and treatment of cardiovascular diseases, chronic inflammation, and tumours; 7-(4-hydroxyphenyl)-1-phenyl-4-hepten-3-one has a therapeutic effect on cancer. Among the active components notably upregulated in ARB, β-estradiol, one of the main phytoestrogen types, can promote the cellular synthesis of DNA, RNA, and various proteins and has potential blood-enriching effects; 11-Deoxy-17-hydroxycorticosterone, a hormone also found in plants, acts as a sterol hormone and has similar functions to β-estradiol [35,36]. pos_10309 was characterised as a potential structural analogue of ramipril; therefore, the angiotensin-converting enzyme (ACE) may be its potential target, which is related to the prevention and treatment of cardiovascular diseases, and erybraedin B is a rosewood compound with potential therapeutic effects against cancer [35]. In the negative ion mode, the log_2_FC of the top 10 differential metabolites ranged from 0.3 to 14, with an average of 4.775 (Supplementary Data Table S4). Among the upregulated active components in ARH, aloin A, malic acid, succinic acid, and L-malic acid have haemostatic effects, and fluoxetine has an antidepressant effect; manumycin A, a microbial-derived antibiotic in the medicinal plant-microbial ecosystem, has an antitumor effect [37]. Among the upregulated active components in ARB, the four dipeptides and S-adenosylmethionine function as nutritional supplementation and physiological improvement [36]; methyl orsellinate has antifungal effects [37]; naringenin-7-O-glucoside exhibits a variety of the aforementioned activities, and its contents in ART, ARB, and ARH thus decreased.

Overall, the top differential metabolites for the ARH, ARB, and ART highlight the tissue-specific characteristics of promoting blood circulation in ART, enriching blood in ARB, and bleeding prevention in ARH. In addition, the material bases of a series of effects such as regulating the female cycle, resisting bacteria, viruses, tumours, and oxidation, enhancing immunity, neuroprotection, and tranquilising nerves were revealed. These findings complement the published information on the metabolic components of Ang root and their pharmacology. The FCs in the top 10 metabolites in the pairwise comparison further showed that ART vs. ARH had the largest difference, ART vs. ARB had the second largest difference and ARH vs. ARB had a relatively small difference (Table 1, Supplementary Data Tables S2–S4); that is, ARB is in a transition state.

### 3.3. Dominant Metabolites in the Different Medicinal Parts of Ang Root and Their Medicinal Efficacy Characteristics

**Figure 3:**
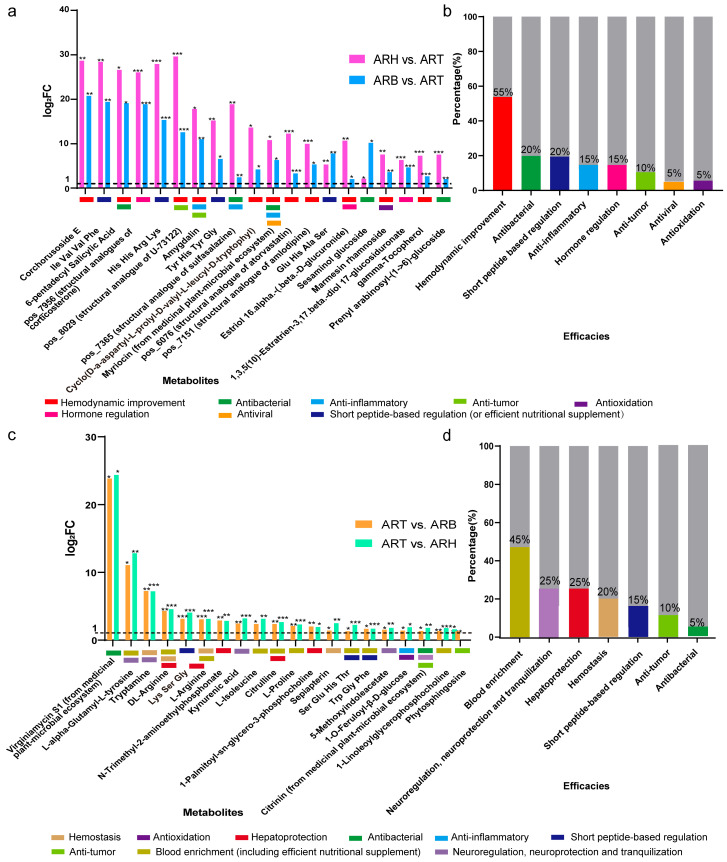
Dominant metabolites (positive ion mode) and corresponding medicinal efficacy patterns in different medicinal parts of Ang root. (**a**,**b**) The top metabolites in ART have higher contents than those in both ARH and ARB, and an analysis of their medicinal efficacy patterns. (**c**,**d**) Top metabolites in both ARH and ARB with higher contents than those in ART and an analysis of their medicinal efficacy patterns. The *, **, and *** denote *p* < 0.1, <0.05 and <0.01, respectively.

Next, we investigated metabolites with absolute superiority in various medicinal parts (Tables S5–S8). These metabolites were defined as upregulated when compared with the other two parts (*p* < 0.05) and when both the log_2_FC and VIP were >1. The metabolites with the highest contents in ART were the most abundant. In the positive ion mode, 135 metabolites were screened, and their contents were higher than those in ARH and ARB simultaneously. The literature and database screening indicated that 79 metabolites were recorded with biological activities [35,36,37] (Supplementary Data Table S5). Regardless of the overall or predominant metabolites, the main efficacy terms of ART were identified as haemodynamic improvement, antibacterial, anti-inflammatory, short peptide-based regulation, and hormone regulation according to the annotation intensity (Supplementary Data Table S5, Figure 3a,b). Among these terms, the components promoting blood circulation were the most commonly observed because 20 types were identified. According to the log_2_FC values, the first 10 blood-activating components included corchorusoside E, 6-pentadecyl salicylic acid, amygdalin, estriol 16.alpha.-(.beta.-D-glucuronide), marmesin rhamnoside, 8-8’-dehydrodiferulic acid, licoleafol, 2,6-dimethoxyquinone, pos_8029 (potential structural analogue of U-73122), pos_6076 (potential structural analogue of atorvastatin), and pos_7151 (potential structural analogue of amlodipine). Second, antibacterial and anti-inflammatory components were annotated to 13 types each. Top antibacterial components with a high FC value included 6-pentadecyl salicylic acid, sesaminol glucoside, prenyl arabinosyl-(1->6)-glucoside, (Z)-1,5-tridecadiene, myriocin (secondary metabolites of fungi in the medicinal plant-microbial ecosystem), and pos_7365 (potential structural analogue of sulfasalazine). We also found that ART was rich in antibiotics with superior content, including myriocin, tobramycin, anisomycin, 3-O-mycarosylerythronolide B, and nystatin, which are presumed to originate from microorganisms in the medicinal plant-microbial ecosystem, indicating that endophytes may have an important influence on the antibacterial efficacy of Ang, based not only on the indirect promotion of the accumulation of active ingredients but also on the direct production of secondary metabolites. Top anti-inflammatory components with high FC value included amygdalin, 5’-prenyllicodione, 8-8’-dehydrodiferulic acid, licoleafol, myriocin (secondary metabolites of fungi in the medicinal plant-microbial ecosystem), and pos_7365 (potential structural analogue of sulfasalazine). Twelve short peptides were identified as dominant metabolites in ART: 10 tetrapeptides and two dipeptides. In addition, steroid hormones with a high content in ART were abundant. Most of these hormones are consistent with the characteristics of phytoestrogens. 

According to the log_2_FC, the first five hormones from the largest to smallest were pos_7956 (potential structural analogues of corticosterone), 11-deoxy-17-hydroxycorticosterone, estriol 16.alpha.-(.beta.-d-glucuronide), 1,3,5(10)-Estratrien-3,17.beta.-diol 17-glucosiduronate, 17-beta-estradiol-3-glucuronide, and 11beta-hydroxyandrost-4-ene-3,17-dione. One of the main functions of Ang is to regulate female menstruation. Intervention with hormones, especially oestrogen, may play an important role in this process [39]. In the negative ion mode, 61 active metabolic compounds were dominant in the ART; more than 20% of them may have the potential to improve the haemodynamics, such as homocitrate, L-anserine, zingerone, cis-9-palmitoleic acid, deoxysappanone B 7,3’-dimethyl ether, myo-inositol, 5-methyltetrahydrofolate, raffinose, salidroside, naringin, neg_5372 (potential structural analogue of bosentan) and neg_5789 (potential structural analogue of lisinopril). The efficacy pattern for the other metabolites was also similar to that of the positive ion mode [35,36,37] (Supplementary Data Figure S4a,b, Supplementary Data Table S6).

ARH and ARB contained few independent dominant compounds. Consequently, most of the attention was allocated to the metabolites with higher levels in the ARH and ARB than in the ART (Supplementary Data Tables S7 and S8). In the positive ion mode, 35 active metabolites were superior to ART for ARH and ARB. The efficacy patterns of these metabolites are concentrated in order of frequency: (1) efficient nutritional supplements, such as various amino acids, dipeptides, and polypeptides; (2) haemostasis, such as tryptamine, L-arginine, and sepiapterin; (3) hepatoprotection, such as N-trimethyl-2-aminoethylphosphonate, 1-palmitoyl-sn-glycero-3-phosphocholine, and L/DL-arginine; and (4) neuromodulation, neuroprotection, and tranquilisation, such as tryptamine, kynurenic acid, and 5-methoxyindoleacetate; and (5) antibacterial, anticancer, immunomodulatory, antioxidation, and other effects, such as 1-O-feruloyl-β-d-glycose, phytosphingosine, and citrinin (secondary metabolites of fungi in a medicinal plant-microbial ecosystem) [35,36,37] (Figure 3c,d, Supplementary Data Table S7). In negative ion mode, the efficacy pattern of active components up-regulated in both ARH and ARB compared with ART was similar to that in positive ion mode (Supplementary Data Figure S4c,d, Supplementary Data Table S8).

According to the efficacy patterns of the dominant compounds in each part, the active ingredients with high contents in each part were consistent with the haemostatic, blood-enriching, and blood-activating effects of RAH, ARB, and ART, respectively. Additionally, ART has advantages in antibacterial, anti-inflammatory, and hormone regulation aspects, and ARH and ARB are inclined to hepatoprotection, neuromodulation, neuroprotection, tranquilisation, and antibacterial effects (Figure 3 and Supplementary Data Figure S4, Supplementary Data Tables S5–S8). Moreover, the haemostatic, blood-enriching, haemodynamics-promoting and other effects of each part were not distinct. By contrast, the differences in metabolites in different parts were reflected in the content of more and less, rather than the presence and absence (Figure 2c, Supplementary Data Figure S3c, Supplementary Data Tables S5–S8). The specific metabolic pattern of any medicinal part reflects this point; for example, in ARB, the quantity and content of the differential metabolites were in a transitional state (Figure 1 and Figure 2, Supplementary Data Figure S3). Specific to some metabolites with a high content of ARB (Supplementary Data Tables S7 and S8), dehydrotremetone was revealed as the active component of nettle, which has anticoagulant and antihypertensive effects. The high content of L-citrulline in the ARB can improve blood indexes and relax blood vessels, lower blood pressure, and maintain normal cholesterol levels [35,36,37]. This intersection of metabolite distributions and efficacy patterns shows that although ARB focused on enriching the blood, it also has a certain efficacy in promoting blood circulation.

### 3.4. Functional Enrichment Patterns of Differential Metabolites

**Figure 4:**
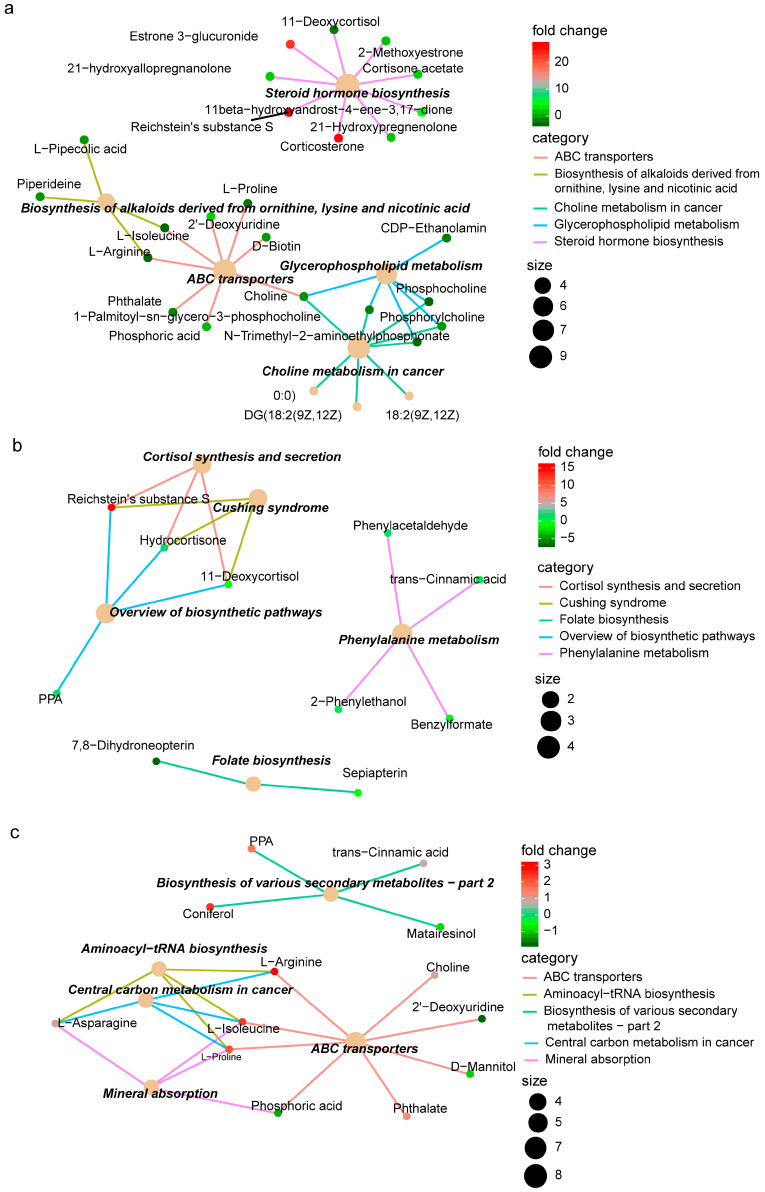
KEGG enrichment analysis of the differential metabolites (positive ion mode) obtained from the different medicinal parts. (**a**–**c**) correspond to ARH vs. ART, ARH vs. ARB, and ART vs. ARB, respectively.

Based on the differential metabolites between the different medicinal parts in Ang roots, KEGG functional enrichment was conducted to further highlight the biological functions that these differential metabolites may affect (Figure 4, Supplementary Data Figure S5). Different medicinal plant parts have different metabolite expressions and functional enrichment patterns. This phenomenon is consistent with the following: the specific metabolite compositions in the parts of Ang root determine the different medicinal efficacies. At the same time, ARH vs. ART was larger than ARH vs. ARB or ARB vs. ART in the differences in the quantity and content of the enriched metabolites. This result is consistent with the aforementioned conclusion that ARH and ART differ in quantity and content, and ARB is in a transitional state.

Regarding the differences in the haemostatic, blood-enriching, and haemodynamics-promoting effects of different parts in Ang root, the comparison results of metabolic enrichment patterns also seem to verify the variation in these efficacies. Specifically, in ART, GABAergic synapse was highly enriched, and most related metabolites were highly expressed (Supplementary Data Figure S5). For example, highly expressed gamma-aminobutyric acid relaxes blood vessels, lowers blood pressure, relieves anxiety, regulates mood, and improves sleep [14]. This finding is consistent with the ART’s efficacy in promoting blood circulation. The functional terms with high enrichment and high expression of related metabolites in ARH were the biosynthesis of alkaloids, folate biosynthesis, and cortisol synthesis and secretion, which were associated with the effects of constricting blood vessels, increasing coagulation factors, and thus a reduced coagulation time [35] (Figure 4, Supplementary Data Figure S5). This functional enrichment pattern is consistent with the haemostatic effects of the ARH. Terms with high levels of enrichment and expression for related metabolites in ARB included alpha-linolenic acid metabolism, vitamin B6 metabolism, mineral absorption, and cortisol synthesis and secretion, which help stimulate bone marrow haematopoietic function, supplement nutrition, and strengthen and improve blood indices (Figure 4, Supplementary Data Figure S5). These effects are consistent with the blood-enriching effects of the ARB.

Furthermore, anticancer effects are one of the most important efficacies of Ang root, and the enrichment of anticancer-related functional terms in various medicinal parts varies. This was mainly reflected in the comparison between ARH and ART. Choline metabolism and central carbon metabolism in cancer were highly enriched with differential metabolites, and the related metabolites were highly expressed in ARH. Central carbon metabolism in cancer was a highly enriched term with differential metabolites, and related metabolites were mainly highly expressed in ARB (Figure 4, Supplementary Data Figure S5). Combining the aforementioned differentially expressed active components, ARH and ARB may have strong antitumour efficacies. Specifically, the relative frequencies of the antitumour-related highly expressed metabolites in ARH or ARB were higher than those in ART (Supplementary Data Tables S2–S8). Although the dominant active ingredients in the ARH and ARB were less than those in the ART, viburtinal, salannin, oleandrin, sepiapterin, guaifenesin, phytosphingosine, 7,8-dihydrofolate, folinic acid, alpha-mangostin, manumycin A (fungal antibiotics attributed to medicinal plant-microbial ecosystem), and citrinin (secondary metabolites of fungi in medicinal plant-microbial ecosystem) were found to have relative or absolute advantages in ARH or ARB and were related to cancer prevention and treatment [35,36,37].

### 3.5. Community Structures of Endophytic Fungi in Ang Root and Distribution Characteristics

**Figure 5:**
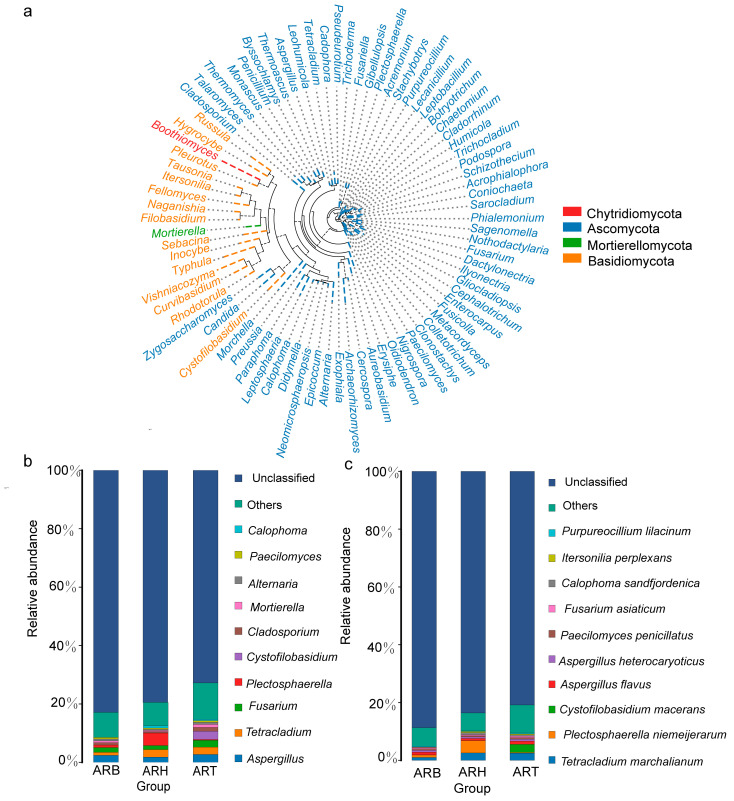
Top endophytic fungi in Ang root with high abundance. (**a**) Phylogenetic analysis at the genus level; (**b**) composition analysis at the genus level; and (**c**) compositional analysis at the species level.

Microbiome analysis based on amplicon sequencing of the fungal ITS identified the largest number of OTUs in the ART samples, followed by the ARB and ARH (Supplementary Data Figure S6). At our sequencing depth, the number of OTUs in the ART, ARB, and ARH was stabilised, and the standard deviation was very small, ensuring the objectivity of the subsequent judgement of species types and relative abundance. Finally, 655 OTUs were obtained and annotated to seven phyla, 21 classes, 53 orders, 109 families, 212 genera, and 245 species. The phylogenetic tree for the top 50 endophytic fungi in Ang root was plotted based on the representative OTU sequences. These fungi belonged to four phyla, among which the Ascomycota and Basidiomycota were the most predominant (Figure 5a). According to the relative abundance, the Top10 genera included *Calophoma*, *Paecilomyces*, *Alternaria*, *Mortierella*, *Cladosporium*, *Cystofilobasidium*, *Plectosphaerella*, *Fusarium*, *Tetracladium*, and *Aspergillus* (Figure 5b). The top 10 species were *Purpureocillium lilacinum*, *Itersonilia perplexans*, *Calophoma sandfjordenica*, *Fusarium asiaticum*, *Paecilomyces penicillatus*, *Aspergillus heterocaryoticus*, *Aspergillus flavus*, *Cystofilobasidium macerans*, *Plectosphaerella niemeijerarum*, and *Tetracladium marchalianum* (Figure 5c).

Species with high abundance levels showed differences among the various medicinal parts. For example, at the genus level, the relative abundance of *Plectosphaerella* was the highest in the ARH group, and that of *Cystofilobasidium*, *Fusarium,* and *Cladosporium* was the highest in the ART group (Figure 5b); among these, *Plectosphaerella* and *Cladosporium* were included in our subsequent differential genera, based on a significance test. At the species level, the relative abundances of *Plectosphaerella niemeijerarum* and *Cystofilobasidium macerans* in the ARH and ART groups, respectively, were the highest (Figure 5c). *Plectosphaerella niemeijerarum* was included in the subsequent differential species by a significance test. Based on a preliminary literature investigation. Among these fungi, species belonging to *Calophoma*, *Paecilomyces*, *Alternaria*, *Cladosporium*, *Plectosphaerella*, *Fusarium*, and *Aspergillus* have been identified as the dominant endophytes in some medicinal plants [21,40]. Many strains contained in *Paecilomyces*, *Alternaria*, *Cladosporium*, *Plectosphaerella*, *Fusarium,* and *Aspergillus* also reportedly produce important bioactive metabolites [39,41]. *Paecilomyces*, *Cladosporium,* and *Plectosphaerella* have significant plant growth-promoting effects [42,43] and are typical biocontrol microbes [42]. Furthermore, for some fungal genera, their strains have also been revealed to be specific plant pathogenic fungi, which may be due to the differences among the various species in the same genus and the large differences in the pathogenicities of the same species in different microecological environments.

### 3.6. Endophytic Fungi with Significant Differences between the Different Medicinal Parts of Ang Root

**Figure 6:**
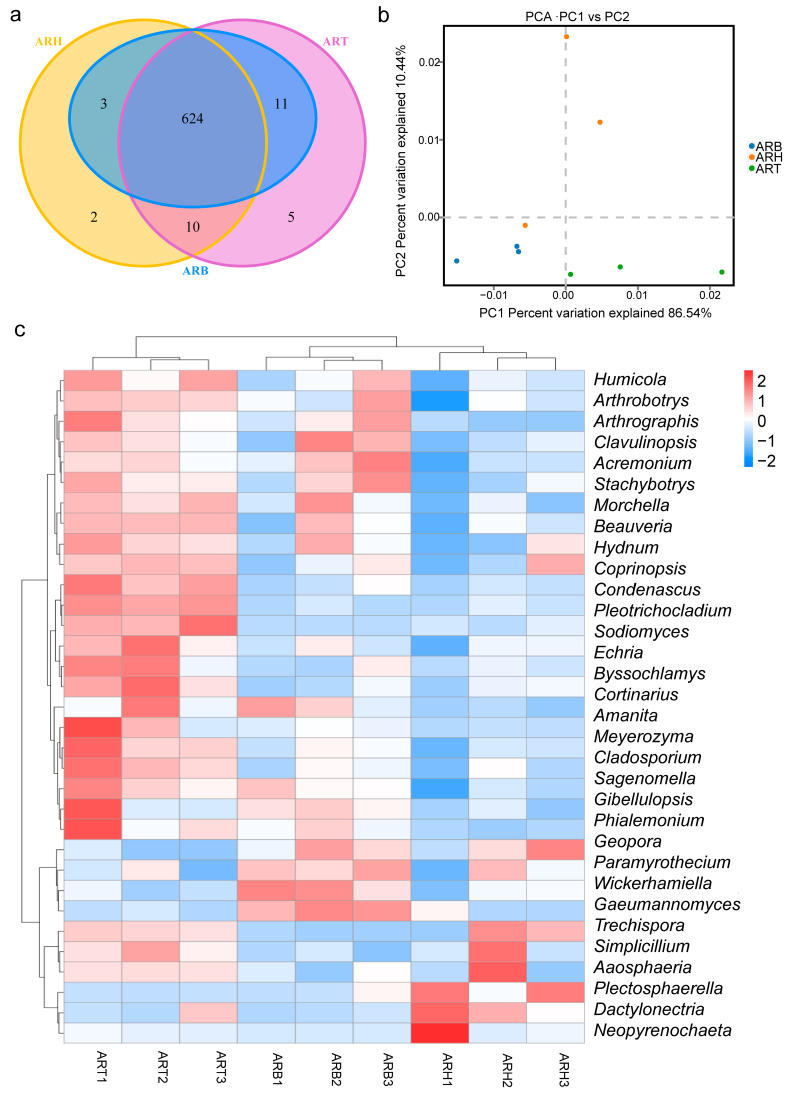
Differential endophytic fungi in the various medicinal parts of Ang root and their distribution characteristics. (**a**) Venn diagram showing the OTUs of the endophytic fungi obtained from the different medicinal parts; (**b**) PCA and clustering of the samples from the three medicinal parts based on the abundance data for the endophytic fungi; (**c**) hierarchical cluster analysis of the samples from the three medicinal parts based on the abundance data for endophytic fungi at the genus level.

The OTU Venn diagram showed that the intersection of fungal OTU in the three medicinal parts reached 624, accounting for a large proportion of all OTUs (95.27%) (Figure 6a). A PCA was performed based on the abundance distribution of OTUs in the ARH, ARB, and ART (Figure 6b). The ARH, ARB, and ART samples were in two-dimensional regions that could be distinguished from each other. This clustering feature indicated objective differences in the fungal communities among the various medicinal parts. Considering the 95.27% intersection of OTUs in different parts, we can conclude that the difference in fungal community structure among ARH, ARB, and ART was mainly in species abundance and not in species richness. Furthermore, the relative abundance of endophytic fungi in the ARH, ARB, and ART groups was compared. Based on variance analysis, 33 genera and 42 species were identified, which showed significant differences among different parts. Regarding the number of differential species, the difference between ART and ARH was the largest, followed by ART and ARB; the difference between ARB and ARH was the smallest (Supplementary Data Figure S7). This is also in accordance with the characteristics of the gradual transition from ART and ARB to ARH. Heatmaps based on the abundance values of the differential species at the genus and species levels showed that more species had higher relative abundances in ART than in ARB and ARH. The difference in species distribution patterns between ART and ARH was the greatest, and ARB was in a transitional state (Figure 6c, Supplementary Data Figure S8). The distributions of endophytic fungi were consistent with the distribution patterns of metabolites in the three medicinal parts.

According to our preliminary literature investigation, ~30% of the aforementioned fungi with significant differences among different medicinal parts were reported as endophytic fungi in medicinal plants [13,14,25], and ~40% were reported to produce bioactive products [22,25,40,44]; biocontrol, growth promotion, and improvement of active component accumulation in the host are also the main characteristics of these endophytic fungi [13,21,43]. This phenomenon indicates that they may be extensively involved in the responses of plants to biotic and abiotic stresses and may enhance the accumulation of bioactive components. Because these endophytic fungi showed significant differences in different medicinal parts, we can infer that they may play an important role in shaping the distribution of active components and the corresponding drug efficacy in different medicinal parts.

### 3.7. Key Endophytic Fungi and Active Components Distributed across Different Medicinal Parts and Their Interactions

Differential metabolic components and endophytic fungi in different medicinal parts of Ang root showed that the difference between ART and ARH was the largest, and ARB was in a transitional state (Figure 2 and Figure 3, Supplementary Data Figure S3, Supplementary Data Tables S2–S8). This is consistent with the spatial distance of the niche and implies a potential relationship between them in the distribution across the medicinal parts. Therefore, correlation analyses and structural equation fitting were used to reveal the interaction further, i.e., quantitatively between differential endophytic fungi and metabolites distributed across medicinal parts, and identify key species and metabolites that are closely related.

**Figure 7:**
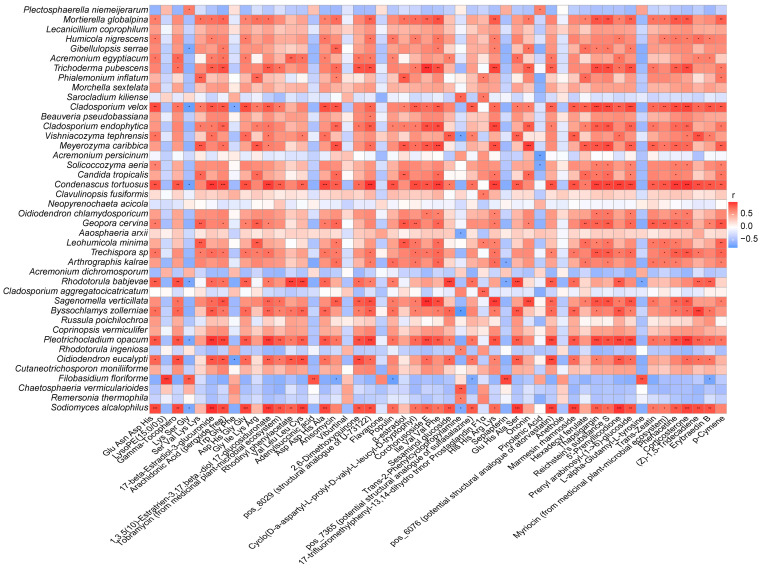
Pearson correlation analysis of the differential metabolites (positive ion mode) and endophytic fungi (species level) based on variations among the different medicinal parts of Ang root. *, **, and *** represent significance levels of 0.1, 0.05, and 0.01, respectively.

Correlation analyses revealed specific endophytic fungi and metabolites with high degrees of correlation. From a microbial perspective, many differential fungi were positively correlated with multiple differential metabolites simultaneously. From a metabolite perspective, many differential metabolites were positively correlated with multiple differential fungi simultaneously (Figure 7, Supplementary Data Figure S9). This correlation topology structure suggests that most differential fungi in different medicinal parts have positive effects on the accumulation of active molecules, and differential fungi affect the expression pattern of metabolites in an organic microecosystem. Conversely, the effects of metabolites on endophytes have a holistic effect based on the interactions among these species.

Some top endophytic fungi positively correlated with multiple metabolites simultaneously may be important species (Figure 7, Supplementary Data Figure S9). According to the number of metabolites significantly positively related to them, the key species were *Condenascus tortuosus*, *Sodiomyces alcalophilus*, *Pleotrichocladium opacum*, *Cladosporium velox*, *Sagenomella verticillata*, *Meyerozyma caribbica*, *Trichoderma pubescens*, *Rhodotorula babjevae*, *Oidiodendron eucalypti*, *Mortierella globalpina*, *Geopora cervina*, *Byssochiamys zollerniae*, and *Filobasidium floriforme*. More than five differential metabolites were significantly and positively related to these fungal species. The fungal species strongly related to the differential metabolites in the positive and negative ion modes were almost identical.

Some important metabolic components and endophytic fungi that are closely related are shown in Figure 7 and Supplementary Data Figure S9. In positive mode, the metabolic components identified for improving blood circulation, including corchorusoside E, arachidonic acid (peroxide-free), 2,6-dimethoxyquinone, pos_6076 (potential structural analogue of atorvastatin), and pos_8029 (potential structural analogue of U-73122), were significantly and positively correlated with *S. alcalophilus*, *P. opacum*, and *S. verticillata*; in addition, except for 2, 6-dimethoxyquinone, the other five components were significantly and positively correlated with *C. tortuosus*. Among phytoestrogens with physiological regulatory activity, 1,3,5(10)-Estratrien-3,17.beta.-diol 17-glucosiduronate and 17-beta-estradiol-3-glucuronide were extremely positively correlated with *C. tortuosus*, *S. alcalophilus*, and *P. opacum*; 11-Deoxy-17-hydroxycorticosterone was extremely positively correlated with *C. tortuosus* and *C. velox*. Prenyl arabinosyl-(1->6)-glucoside, a glucoside with antibacterial activity, was extremely and positively correlated with *C. tortuosus*, *P. opacum*, and *C. velox*. Among the other important active components, 5’-prenyllicodione, erybraedin B, γ-tocopherol, p-cymene, β-estradiol, marmesin rhamnoside, and anisomycin (antibiotics produced by fungi in medicinal plant-microbial ecosystem) were significantly positively correlated with more than three endophytic fungal species, most of which were the top species aforementioned, showing a strong correlation with differential metabolites (Figure 7). In negative ion mode, the important active metabolic components, including genkwanin, 2’-O-methylguanosine, 3-methyluridine, sappanone A dimethyl ether, and neg_1428 (potential structural analogue of zidovudine), were extremely positively correlated with two or three of *C. tortuosus*, *S. alcalophilus*, *P. opacum*, *C. velox*, and *O. eucalypti*. Among the other important metabolites, homocitrate, oxoglutaric acid, (±)7-epi jasmonic acid, (-)-riboflavin, vidarabine (antibiotics produced by *Streptomyces* in the medicinal plant-microbial ecosystem), and neg_5789 (potential structural analogue of lisinopril) were significantly positively correlated with more than three endophytic fungal species, and most of them were the top species mentioned aforementioned, with a strong correlation with differential metabolites (Supplementary Data Figure S9). Overall, a significant positive correlation between the active components with important medicinal effects and various key endophytic fungi was presented quantitatively and visually, and this provides an important reference for the microecological regulation, microbial transformation, and biosynthesis of active metabolites in CHM.

**Figure 8:**
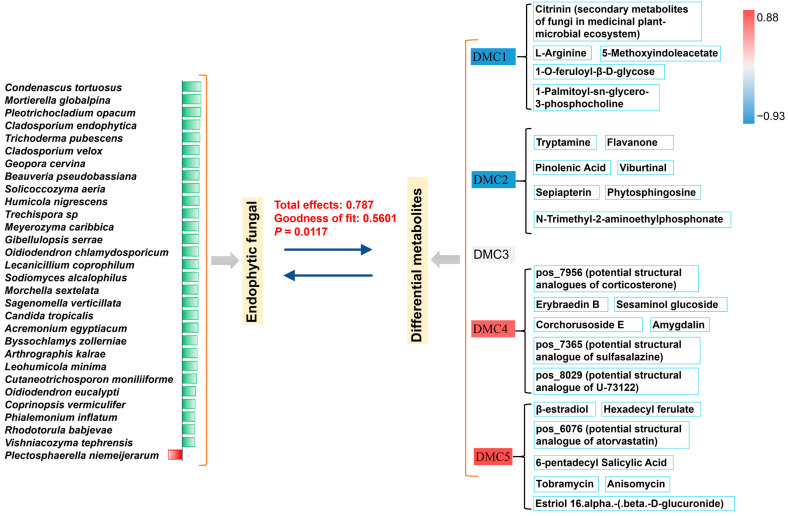
Structural equation model for the endophytic fungi (species level) and metabolites (positive ion mode) based on the differences among the medicinal parts of Ang root. The green bar chart shows the indication values for the differential species to the microbial module; the red-blue colour gradation shows the indicator values for the differential metabolite clusters to the metabolite module.

The dimensionality of the metabolite content data was reduced during structural equation fitting to ensure the comprehensiveness of the metabolite information and simplify the calculation. All differential metabolites were clustered hierarchically according to the distribution patterns in different medicinal parts, and the average abundance of each cluster group in each part was used for model fitting. The total influence values of differential endophytic fungi on differential metabolites in the positive and negative ion modes were 0.787 and 0.796, respectively, and the goodness of fit of the corresponding models was approximately 0.56. The *p*-value was less than 0.05 (Figure 8, Supplementary Data Figure S10). These parameters quantitatively indicated a significant correlation between the endophytic fungi and metabolites. For the microbial module, the fungal species with loading values greater than 0.6 for the whole differential fungi were 30 and 29 in the fitting with the metabolites of positive and negative ion modes, respectively, accounting for 70% and 58% of all differential fungal species (42) (Figure 8, Supplementary Data Figure S10). For the metabolic module, among the five differential metabolite clusters in the positive ion mode (defined as DMC1–5), all the absolute loading values of DMC1, DMC2, DMC4, and DMC5 for all metabolites were greater than 0.8, and the value of DMC3 was the smallest at approximately 0.5 (Figure 8). However, most of the important top differential metabolites belonged to DMCs with high loading values, and DMC3 contained few of the top differential metabolites identified. Similar fitting results were obtained in negative ion mode (Supplementary Data Figure S10). Thus, important differential metabolites contribute to the close relationship between the metabolite module and microbial module and further highlight the potential mutual shaping of endophytic fungi and important bioactive substances in different medicinal parts.

## 4. Discussion

### 4.1. Supplement Showing Distribution Map of Active Compounds in Different Medicinal Parts of Ang Root

Because of the substantial potential of Ang as a natural product bank, the chemical basis of its broad-spectrum efficacy should be deeply explored. The medicinal differences among different Ang root parts are widely recognised in clinical Chinese medicine [5]. However, the metabolic bases for the efficacy difference across ARH, ARB, and ART remain unclear. In terms of curative effects on the cardiovascular system, the dominant metabolites of the different medicinal parts revealed in this study further verified the advantages of ARH, ARB, and ART in terms of haemostasis, blood enrichment, and blood flow improvement at the molecular level, respectively. Specific natural compounds that perform the corresponding efficacies were also highlighted. For example, sepiapterin, sloin A, and malic acid, which are highly expressed in ARH, and L-alpha-glutamyl-L-tyrosine, tryptamine, and L-arginine, which are highly expressed in both ARH and ARB, have been revealed to have haemostatic effects. L-isoleucine, L-proline, 1-linoleoylglycerophosphocholine, L-tryptophan, L-tyrosine, folinic acid, 7, 8-dihydrofolate, nicotinamide adenine dinucleotide (NAD), L-lysine monohydrochloride, and L-citrulline, which were highly expressed in ARH and ARB and have potential blood-enriching effects as nutrients or coenzymes that promote biosynthetic reactions. Metabolites, such as corchorusoside E, 6-pentadecyl salicylic acid, amygdalin, estriol 16.alpha.-(.beta.-D-glucuronide), marmesin rhamnoside, gamma-tocopherol, 8-8′-dehydrodiferulic acid, licoleafol, 2,6-dimethoxyquinone, arachidonic acid (peroxide-free), homocitrate, L-anserine, zingerone, cis-9-Palmitoleic acid, deoxysappanone B 7,3’-dimethyl ether, myo-inositol, raffinose, salidroside, naringin, pos_8029 (potential structural analogue of U-73122), pos_6076 (potential structural analogue of atorvastatin), pos_7151 (potential structural analogue of amlodipine), and neg_5372 (potential structural analogue of bosentan), which are highly expressed in ART, are potential naturally active ingredients that promote blood circulation [35,36,37].

Notably, our results also quantitatively showed that the efficacies of ARH, ARB, and ART are not strictly defined but intersect. These characteristics are reflected in three aspects: most metabolites are present in different contents in different parts, rather than absolute presence or absence; a small number of metabolites were highly expressed in one part but indicate the representative efficacy of another part; and natural compounds with antibacterial, anti-inflammatory, antiviral, antitumour, and depression-relieving effects have been identified in all medicinal parts of Ang root [35,36,37]. For example, kynurenic acid, 5-methoxyindoleacetate, 1-O-feruloyl-β-D-glucose, phytosphingosine, alpha-mangostin, S-(1, 2-dicarboxyethyl) glutathione, folinic acid, 7, 8-dihydrofolate, virginiamycin S1 (antibiotics produced by *Streptomyces* in the medicinal plant-microbial ecosystem), and citrinin (Secondary hydrolysates of fungi in a medicinal plant-microbial ecosystem) are highly expressed in ARH and ARB, and 6-pentadecyl salicylic acid, thapsigargin, amygdalin, 5’-prenyllicodione, 4-isopropylbenzyl alcohol, sesaminol glucoside, marmesin rhamnoside, sappanone A dimethyl ether, L-anserine, zingerone, deoxysappanone B 7,3’-dimethyl ether, coumarin, salidroside, naringin, myriocin (secondary metabolites of fungi in medicinal plant-microbial ecosystem), pos_7365 (potential structural analogue of sulfasalazine), and zidovudine (neg_1428 (potential structural analogue of zidovudine) are highly expressed in ART.

Comparative metabolomics revealed the tissue-specific natural active components and further clarified material bases of the different indications for various medicinal parts, supplementing the pharmacology of Ang. Moreover, these metabolic components corresponding to important efficacies are key to the modern application of natural compound libraries in Ang. Similar to how artemisinin was extracted from *Artemisia annua* in the 1970s and identified as a natural compound for treating malaria, the chemical synthesis, biosynthesis, and drug development of artemisinin were greatly promoted.

### 4.2. Spatial Distribution Patterns and Interaction Mechanisms of Endophytic Fungi and Active Metabolites

Endophytic fungi have been widely revealed to shape the quality of medicinal plants based on plant-microbe interactions [12,22]. In this study, most of the significantly differential species were identified as endophytic fungi of Ang for the first time, although several of them have been reported as endophytic fungi of other medicinal plants [12,21]. These endophytic fungi possess important characteristics, including promoting growth, producing or transforming drug-active components, resisting biotic and abiotic stresses, inducing plant immunity, changing host epigenetics, and exerting biocontrol effects [42,44]. This suggests that their differential distribution across the medicinal parts has an important effect on each part’s accumulation pattern of active components.

Understanding the potential mechanisms underlying the distribution of active ingredients in medicinal plants across tissues is important. From a genetic perspective, the tissue specificity of metabolic components is determined by genetic differentiation [9], and from an environmental perspective, different parts are influenced by specific spatiotemporal factors and the microenvironment created by them [8,19]. On this basis, the potential role of the cross-tissue distribution of endophytic fungi in determining the distribution of active components in different medicinal parts of Ang root remains to be elucidated. This study quantitatively and visually presented the distribution patterns of endophytic fungi and metabolites in different medicinal parts of Ang root. The results showed that the ARH, ARB, and ART transition patterns were synchronised between endophytes and metabolites. Regardless of the endophytic fungal species or metabolic components, the difference was the largest for ART vs. ARH, compared with that of ART vs. ARB and ARH vs. ARB, and ARB was in a transitional state.

Moreover, more dominant metabolic components and fungal species were found in ART than in ARH and ARB. Structural equation and correlation analyses further quantitatively revealed significant correlations between active metabolites and fungal species. According to the quantitative fitting results, the influence of microbial modules on metabolic modules is very high; most of the differential endophytic fungi have high indicative values for microbial modules, and important differential metabolic components have high indicative values for metabolite modules; many dominant endophytic fungi have a very significant positive correlation with the active metabolic components. Therefore, from the dual perspectives of biological phenomenon observation and mathematical model fitting, our research highlights that the differential distribution of endophytic fungi in different Ang root parts has a potential shaping effect on the cross-parts distribution pattern of metabolic components. Although external temporospatial factors should be considered, the specific microenvironment, comprising the specific metabolite patterns in different plant parts, may affect the tissue part-specific distribution of endophytic fungi [15,16]. Different tissue parts recruit specific microbial species based on their metabolic components or exert influence through quorum sensing, shaping specific microbial community structures. For example, amino acids and polypeptides were generally identified; they have many physiological functions (e.g., nutritional supplementation, immune regulation, and metabolism improvement) and are important microbial regulatory factors.

Additionally, many endophytic fungi were positively correlated with many metabolites simultaneously. For example, *C. tortuosus*, *S. alcalophilus*, *P. opacum*, and other microbial species are positively correlated with many important metabolic components simultaneously. Relevant metabolic components have valuable biological activities, including haemodynamics-promoting, anti-inflammatory, antibacterial, antiviral, and anticancer properties [35,36,37]. These results indicate that these important endophytic fungi can induce plants to produce or directly synthesise abundant active secondary metabolites or have broad-spectrum biotransformation abilities. This result is consistent with those of our literature review on the differential endophytic fungi detected [12,13,21]. Conversely, many metabolites were positively correlated with many endophytic fungi. This result indicates that the role of endophytic fungi in promoting the accumulation of active components is related to the synergistic effect of communities formed by various microbial species and that any metabolic component regulates the microbiota at the community level based on the interaction between species. Therefore, our results highlight that the interaction between endophytic fungi and metabolites distributed across tissue sources is bidirectional and pluralistic. Many metabolites are positively related to many microorganisms simultaneously and vice versa; they cross and influence each other. Overall, this two-way and multidimensional complex interaction between endophytic fungi and metabolic components of Ang root may further shape their closely related cross-part distribution patterns.

### 4.3. Important Research and Application Values of Key Metabolites and Endophytic Fungi Closely Related across Adjacent Medicinal Parts

In contrast with the literature, which focused on comparing different tissues with far spatial distances, such as roots, stems, and leaves, this study strictly compared the adjacent medicinal parts of Ang root and revealed the distribution characteristics of their metabolic components and endophytic fungi and the potential relationship between the two [8]. Endophytes play important roles in promoting the accumulation of active components in the host. In this study, several endophytic fungal species with significant positive correlations with many important active components were identified. These have important reference values for regulating the accumulation of active components in Ang through microecology. Endophytes often produce secondary metabolites with structures similar to those of their hosts. This ability is due to the interaction of genes between endophytic fungi and the host in the long-term evolution process, or the endophytic fungi using host active molecules as substrates for microbial transformation and producing derivatives [12,13,21]. Therefore, endophytes are important repositories of valuable natural products and relevant genes.

Regarding the discovery of synthetic genes of natural active products, based on the obvious variation in metabolic components between the neighbouring medicinal parts of Ang root revealed in this study, it is expected to help focus on some gene sets that determine differential metabolites by combining transcription and post-transcription information among various medicinal parts of Ang root. Therefore, the spatially specific distribution map of endophytic fungi and active metabolites in Ang root and their potential relationships revealed in this study are significant for the biosynthesis of active metabolites of Ang. Furthermore, some metabolites with obvious advantages in any medicinal part remain unrevealed in pharmacology, and some undisclosed endophytes associated with important active ingredients, the relationship between them and the accumulation of medicinally active ingredients, the synthesis of secondary metabolites, and the mechanism of microbial transformation require further research. This study lays a foundation for these relevant studies.

## 5. Conclusions

This study established an information database for metabolites and endophytic fungi across adjacent medicinal parts of Ang root and systematically investigated the differences and correlations in their distributions. The results highlight the bidirectional and multivariate complex interactions between endophytes and their host metabolites, contributing to their closely related differential distribution patterns across tissues. Simultaneously, closely related microbes and their metabolic components were also identified. These results provide reference data that help regulate the quality of Ang through endophytic inoculation. Furthermore, these data help in mining Ang or its endophytes-derived gene clusters related to the synthesis of natural active products.

## Figures and Tables

**Table 1:** The most up- and down-regulated metabolites between the medicinal parts of Ang root (ARH vs. ART) in the positive ion mode and their efficacy annotations based on published literature and existing databases.

Metabolites	log_2_FC	*p*	VIP	Efficacies
Up-regulated in ART				
pos_8029 (potential structural analogue of U-73122)	29.619735	0.000009	1.548186	A&J
Corchorusoside E	28.693819	0.001097	1.429212	A
Ile Val Val Phe	28.417091	0.006163	1.290369	B&F
His His Arg Lys	27.949976	0.000712	1.446415	B&F
11-Deoxy-17-hydroxycorticosterone	26.934995	0.003347	1.341104	H
6-pentadecyl salicylic acid	26.593057	0.011570	1.223086	A&D
pos_7956 (potential structural analogues of corticosterone)	26.034194	0.000350	1.465804	H
Thapsigargin	23.260326	0.002925	1.351772	L
Ononin	18.885931	0.041072	1.101388	A&D&E&K&J
Hexadecyl ferulate	18.372359	0.024347	1.037334	A
Up-regulated in ARH				
Virginiamycin S1	24.388477	0.013138	1.186106	D
2’-O-methylcytidine	18.113741	0.026446	1.086260	O/U
beta-Nicotinamide D-ribonucleotide	15.582520	0.000222	1.485885	O/U
6,7-Dimethoxyisoquinoline	12.977668	0.018082	1.170311	O/U
L-alpha-Glutamyl-L-tyrosine	12.822422	0.001699	1.410215	B&F&I
Anethole	12.567253	0.012978	1.204438	B
7-(4-Hydroxyphenyl)-1-phenyl-4-hepten-3-one	10.474889	0.000054	1.525504	D&J
Viburtinal	9.307067	0.029676	1.093222	C&E&I&J
Tryptamine	7.205199	0.000005	1.524918	C
Nalpha-Methylhistidine	6.841870	0.019508	1.133064	O/U

## Data Availability

All data generated or analysed during this study are included in this published article.

## Supplementary Materials

The following supporting information can be downloaded at: https://www.mdpi.com/article/10.3390/metabo14010048/s1, Figure S1: Peaks of all QC sample outputs determined using the LC-MS system in negative ion mode; Figure S2: OPLS-DA for the samples from different medicinal parts based on metabolite abundance data; Figure S3: Analysis of the differential metabolites between pairs of medicinal parts in Ang root (ARH vs. ART, ARH vs. ARB, and ART vs. ARB) under a negative ion mode; Figure S4: Dominant metabolites (negative ion mode) and corresponding medicinal efficacy patterns in different medicinal parts of Ang root; Figure S5: KEGG enrichment analysis of the differential metabolites (negative ion mode) obtained from the different medicinal parts; Figure S6: Relationships between the ITS sequence numbers and the OTU numbers in the microbiome analyses for the different medicinal parts of Ang root; Figure S7: Venn diagram showing the endophytic fungi at the genus (a) and species (b) levels in different medicinal parts of Ang root; Figure S8: Hierarchical cluster analysis of the samples from the different medicinal parts based on the abundance data for endophytic fungi at the species level; Figure S9: Pearson correlation analysis of the differential metabolites (negative ion mode) and endophytic fungi (species level) based on variations among the different medicinal parts of Ang root; Figure S10: Structural equation model for the endophytic fungi (species level) and metabolites (negative ion mode) based on the differences among the medicinal parts of Ang root; Table S1: Classification of the Ang root metabolites identified using untargeted metabolomics; Table S2: The most up- and down-regulated metabolites between medicinal parts of Ang root (ARH vs. ART) in the negative ion mode and their efficacy annotations based on published literature and existing databases; Table S3: The most up- and down-regulated metabolites between medicinal parts of Ang root (ARB vs. ART) in the positive and negative ion mode and their efficacy annotations based on published literature and existing databases; Table S4: The most up- and down-regulated metabolites between medicinal parts of Ang root (ARH vs. ARB) in the positive and negative ion mode and their efficacy annotations based on published literature and existing databases; Table S5: Metabolites (positive ion mode) in ART with superior content over both ARH and ARB, and annotation of their efficacy; Table S6: Metabolites (negative ion mode) in ART with superior content over both ARH and ARB, and annotation of their efficacy; Table S7: Metabolites (positive ion mode) in ARH and ARB with superior content over ART, and annotation of their efficacy; Table S8: Metabolites (negative ion mode) in ARH and ARB with superior content over ART, and annotation of their efficacy.
