# Supplementary material for: Longitudinal Distribution Map of the Active Components and Endophytic Fungi in Angelica sinensis (Oliv.) Diels Root and Their Potential Correlations
Source: Metabolites. 2024 Jan 12;14(1):48. doi: 10.3390/metabo14010048 (PMC10821059; doi:10.3390/metabo14010048)
Supplement: Supplementary file 1 [file metabolites-14-00048-s001.zip › metabolites-2787695-supplementary.pdf]

Supplementary data

Longitudinal Distribution Map of the Active Components and Endophytic Fungi in *Angelica sinensis* (Oliv.) Diels Root and Their Potential Correlations

Ying Sun [1,†], Rong Guo [2,†], Yuting Geng [3,†], Hushan Shang [4], Xiaopeng Guo [1,2,*], Yue Wu [1], Yonggang Wang [2,*], Li Li [1], Xuee Li [2], Shengli Zhang [2], Ning Xu [1] and Xueyan Li [2]

[1] Gansu Institute for Drug Control, Lanzhou 730070, China
[2] School of Life Science and Engineering, Lanzhou University of Technology, Lanzhou 730050, China
[3] Department of Water Resources of Gansu Province, Lanzhou 730020, China
[4] Dingxi Academy of Agricultural Sciences, Dingxi 743002, China
* Correspondence: guoxp@lut.edu.cn (X.G.); wangyg@lut.edu.cn (Y.W.); Tel.: +86-15101200201 (X.G.); +86-13679492830 (Y.W.)
† These authors contributed equally to this work.

Supplementary data

Tab. S1 Classification of the Ang root metabolites identified using untargeted metabolomics, including metabolites obtained from positive and negative ion modes.

SuperClass	HMDB taxonomy	Metabolites number		
		Pos	Neg	Total
Organoheterocyclic compounds	Heteroaromatic compounds	21	12	33
	Indoles and derivatives	13	15	28
	Pteridines and derivatives	15	11	26
	Isocoumarans	23	0	23
	Pyridines and derivatives	20	3	23
	Biotin and derivatives	22	0	22
	Imidazodiazepines	19	0	19
	Pyrrolizidines	18	0	18
	Benzopyrans	17	0	17
	Dihydrofurans	0	17	17
	Furofurans	0	16	16

	Lactams	14	0	14
	Imidazopyrimidines	6	4	10
	Quinolines and derivatives	0	7	7
	Benzofurans	4	0	4
	Benzodioxoles	3	0	3
	Furans	0	2	2
	Oxazinanes	2	0	2
	Diazinanes	1	0	1
	Lactones	0	1	1
Lipids and lipid-like molecules	Steroids and steroid derivatives	60	22	82
	Glycerolipids	37	26	63
	Prenol lipids	51	0	51
	Fatty Acyls	33	16	49
	Sphingolipids	0	27	27
	Glycerophospholipids	14	0	14
Organic acids and derivatives	Carboxylic acids and derivatives	34	34	68
	Hydroxy acids and derivatives	35	2	37
	Iivatives	0	36	36
	Keto acids and derivatives	0	35	35
Benzenoids	Benzene and substituted derivatives	25	4	29
	Phenol ethers	28	0	28
	Indanes	27	0	27
	Phenol esters	26	0	26
	Naphthalenes	10	8	18
	Phenols	6	7	13
	Anthracenes	0	9	9
Phenylpropanoids and polyketides	Coumarins and derivatives	16	4	20
	Kavalactones	17	0	17
	Cinnamic acids and derivatives	7	9	16
	Isoflavonoids	15	0	15
	Phenylpropanoic acids	14	0	14
	Cinnamyl alcohols	0	11	11
	Cinnamaldehydes	9	1	10
	Flavonoids	0	10	10
	Macrolides and analogues	0	2	2
Nucleosides, nucleotides, and analogues	Pyrimidine nucleosides	8	7	15
	5'-deoxyribonucleosides	0	8	8
	Purine nucleosides	4	4	8
	Pyrimidine nucleotides	5	0	5
	Pyridine nucleotides	3	0	3
	Purine nucleotides	2	0	2
	Ribonucleoside 3'-phosphates	1	0	1
Organic oxygen compounds	Organooxygen compounds	15	17	32
Organic nitrogen compounds	Organonitrogen compounds	7	0	7
Homogeneous non-metal compounds	Non-metal oxoanionic compounds	1	1	2
Alkaloids and derivatives	Ergoline and derivatives	1	0	1

Lignans, neolignans and related compounds	Furanoid lignans	1	0	1

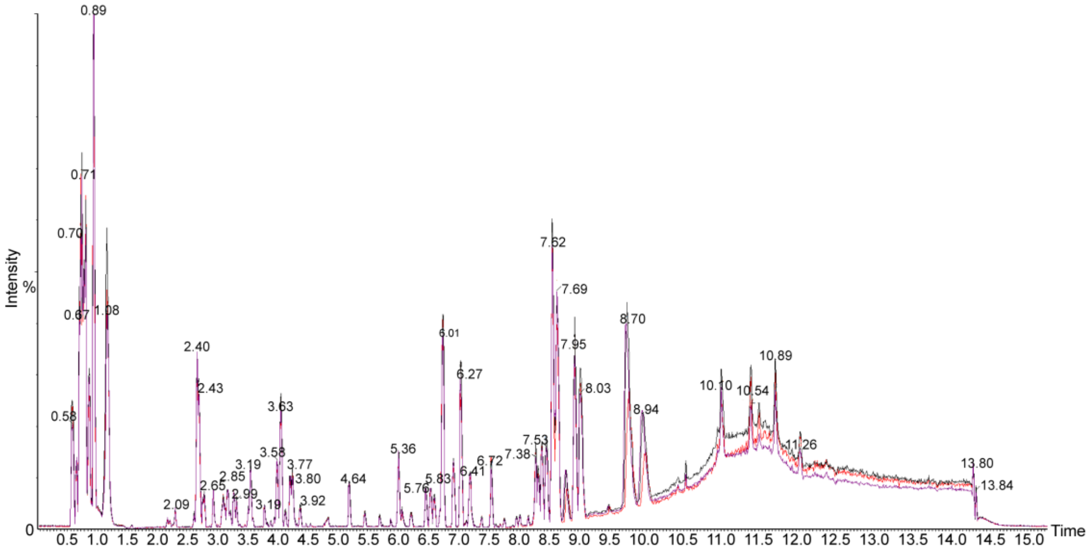

Fig. S1 Peaks of all QC sample outputs determined using the LC-MS system (negative ion mode) almost overlap, indicating the stability of the system.

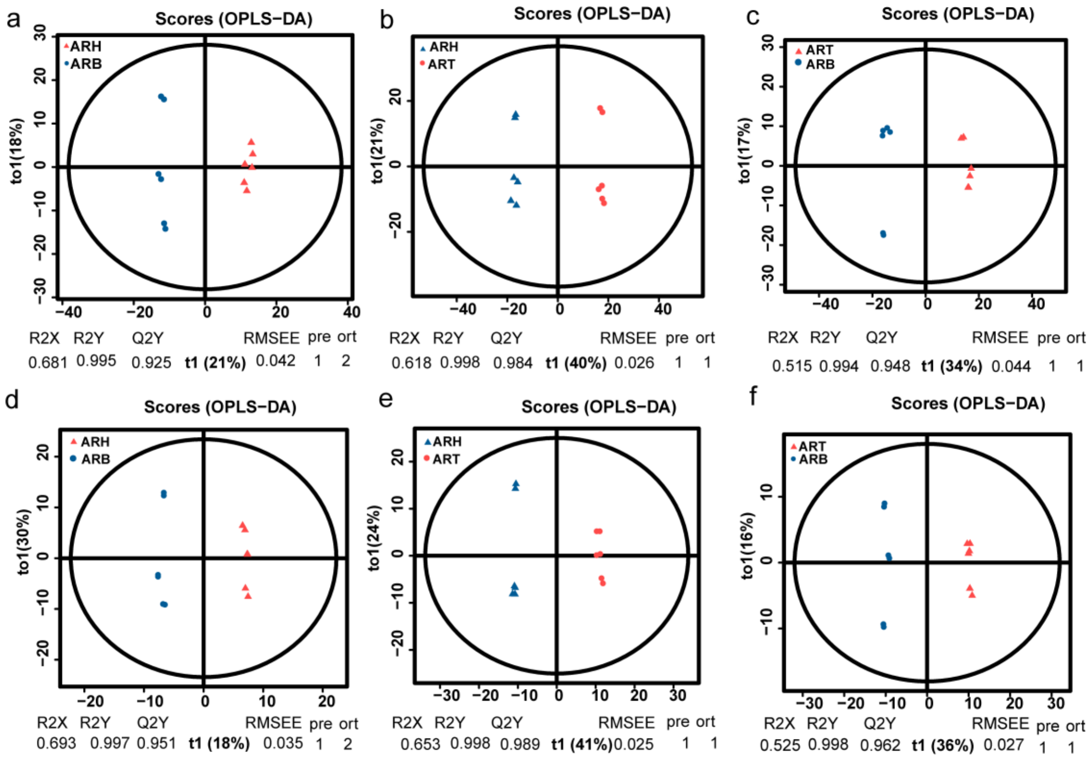

Fig. S2 OPLS-DA for the samples from different medicinal parts based on metabolite abundance data. Fitting results of the discriminant model according to the metabolites identified in the positive ion mode (a, b, and c), and the negative ion mode (d, e, and f).

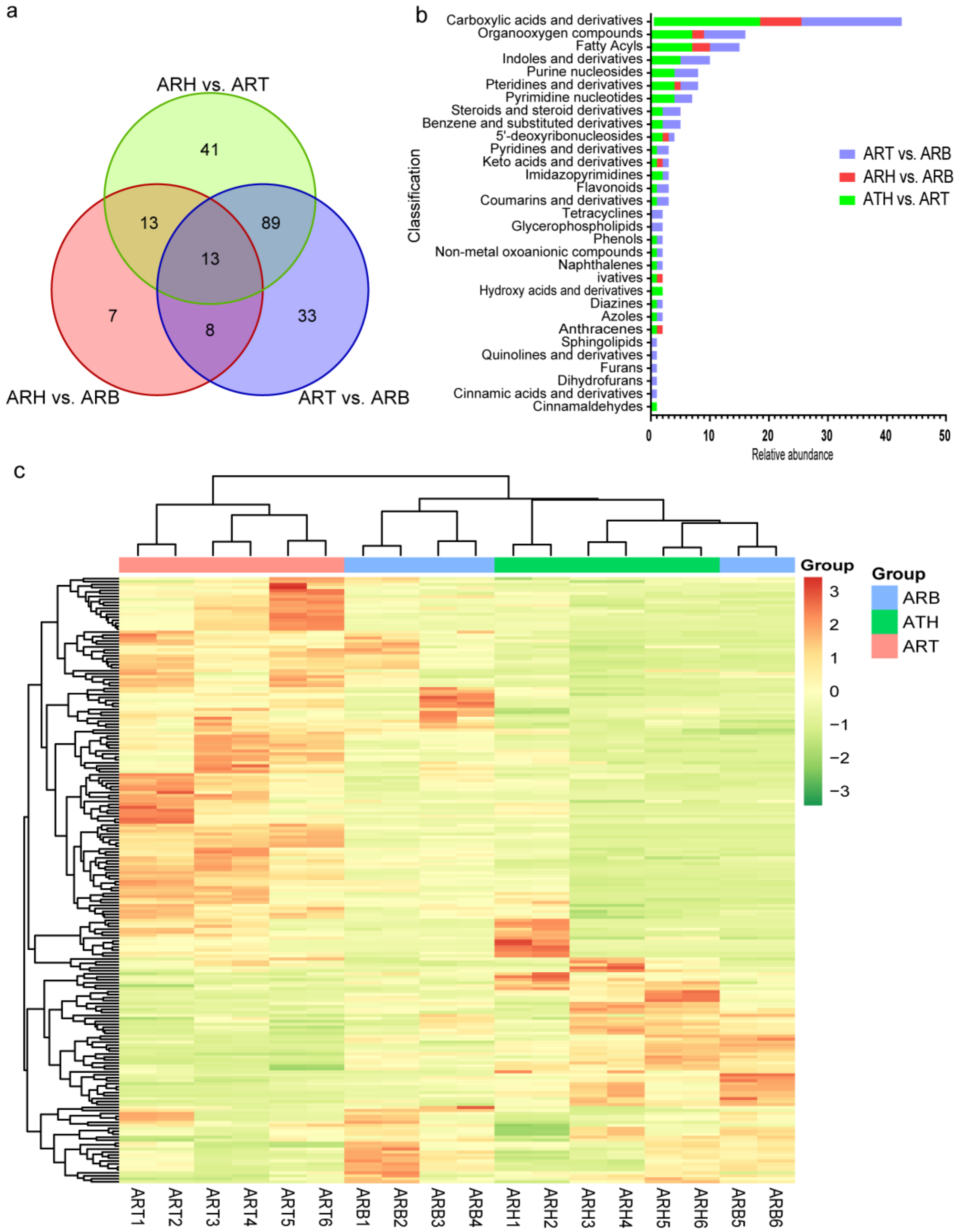

Fig. S3 Analysis of the differential metabolites between pairs of medicinal parts in Ang root (ARH vs. ART, ARH vs. ARB, and ART vs. ARB) under a negative ion mode. a, Venn diagram showing the differential metabolites obtained by pairwise comparison; b, categorical distributions of the differential metabolites in the pairwise comparisons; c, hierarchical clustering of samples from different medicinal parts based on the abundance data for the differential metabolites.

Tab. S2 The most up- and down-regulated metabolites between medicinal parts of Ang root (ARH vs. ART) in the negative ion mode and their efficacy annotations based on published literature and existing databases.

Metabolites	$\log_2$ FC	*P*	VIP	Efficacies
Up-regulated in ART				
(±)7-epi Jasmonic Acid	24.05605131	0.01933689	1.114826215	O/U
Levofloxacin	21.120608	0.010691571	1.189012861	D
Naringenin-7-O-Glucoside	20.97447691	0.026300213	1.108350735	A&J&K
Procyanidin C1	20.43911118	0.035547733	1.082350051	K
(E)-2-Butenyl-4-methyl-threonine	20.35408852	0.00336545	1.330190291	O/U
Zidovudine	18.52287241	0.000328323	1.473882235	L
Sappanone A Dimethyl Ether	18.37546152	3.22997E-07	1.544781928	E&I&K
Genkwanin	18.01104845	0.005347421	1.277507851	E
D-Quinovose	17.37229918	0.009625896	1.256741666	E&J
Compactin	17.30075744	0.009613316	1.233847214	A&D
Up-regulated in ARH				
L-Citrulline	17.06407765	0.021322857	1.136057815	B
Oleandrin	16.23242615	0.001554801	1.395790335	J&L
Hygromycin B	15.77239257	0.013552818	1.151534867	D
Salannin	11.1168727	0.040288286	1.016505585	D&J&L
Androsterone sulfate	11.02829728	6.04545E-07	1.530880806	H
D-Glycerate 2-phosphate	10.94182852	5.20276E-05	1.499242064	O/U
(±)Abscisic Acid	7.921034323	0.008287957	1.252012849	O/U
Manumycin A	7.068500558	0.007207878	1.270556307	D&J
Glutathione, oxidized	5.925103791	0.000430685	1.468147424	K
L-Lysine monohydrochloride	2.882769875	0.00109482	1.408420762	B&C

Note: The Capital letters represent the efficacy items of metabolites, which are as follows: A, Hemodynamic improvement; B, Efficient nutritional supplement; C, Hemostasis; D, Antibacterial; E, Anti-inflammatory; F, Short peptide-based regulation; G, Hepatoprotection; H, Hormone regulation; I, Neuromodulation, neuroprotection and tranquilization; J, Anti-tumor; K, Antioxidation; L, Antiviral. The 'O/U' was used to indicate other or unclear efficacies.

Tab. S3 The most up- and down-regulated metabolites between medicinal parts of Ang root (ARB vs. ART) in the positive and negative ion mode and their efficacy annotations based on published literature and existing databases.

Metabolites	log_2 FC	*P*	VIP	Efficacies
Up-regulated in ART (positive ion mode)				
20-hydroxy-PGF2a	25.418283	0.017170	1.327444	A
Isoleucyl-Histidine	21.614999	0.009634	1.366361	B&F
Corchorusoside E	20.759734	0.001139	1.509182	A
Ile Val Val Phe	19.419996	0.008975	1.248720	B&F
6-pentadecyl Salicylic Acid	19.109225	0.012289	1.351436	A&D
pos_7956 (potential structural analogues of corticosterone)	18.890987	0.000355	1.565330	H
Echinacoside	15.990446	0.049407	1.063720	E&I&K
His His Arg Lys	15.350677	0.000551	1.448035	B&F
Mitomycin	15.288801	0.013661	1.319412	J
Thapsigargin	15.211655	0.003325	1.407567	E&J
Up-regulated in ART (negative ion mode)				
Gentiobiose	23.654271	0.034234	1.101296	O/U
Genkwanin	20.774493	0.005498	1.354924	E
Gln Asn Glu Glu	19.224167	0.025391	1.145099	B&F
Flavin mononucleotide (FMN)	17.964504	0.023795	1.171169	O/U
2-Ethyl-4-hydroxy-5-methyl-3(2H)-furanone	14.916657	0.042682	1.057640	O/U
Glutamine	14.837699	0.028751	1.121713	E
Levofloxacin	14.113105	0.011600	1.264487	D
2'-O-methylguanosine	13.583382	0.000001	1.626394	O/U
7-Methylguanosine	12.575918	0.001684	1.318273	O/U
Tetracycline	11.878423	0.006361	1.337141	O/U
Up-regulated in ARB (positive ion mode)				
Virginiamycin S1	23.864641	0.019134	1.230527	D
2'-O-methylcytidine	18.318932	0.000218	1.604279	O/U
L-alpha-Glutamyl-L-tyrosine	11.076554	0.012707	1.291315	B&F&I
Tryptamine	7.249718	0.005405	1.406326	C&I

Nalpha-Methylhistidine	6.446779	0.023545	1.136743	O/U
DL-Arginine	4.304115	0.003394	1.455654	B&C&G
DL-Benzylsuccinic acid	3.959036	0.012056	1.353680	O/U
Lys Ser Gly	3.762833	0.009348	1.346463	B&F
Homatropine	3.275559	0.002159	1.481089	O/U
L-Arginine	3.089285	0.000000	1.659133	B&C&G
Up-regulated in ARB (negative ion mode)				
L-Citrulline	18.047198	0.008253	1.336018	B
D-Glycerate 2-phosphate	10.027066	0.001245	1.445770	O/U
Salannin	8.560896	0.043717	1.066953	D
(±)Abscisic Acid	6.152383	0.008421	1.275040	O/U
Homogentisic acid	5.954816	0.004510	1.295302	O/U
2,5-Dihydroxybenzoic acid	2.377378	0.006804	1.321143	O/U
Nicotinamide adenine dinucleotide (NAD)	2.338834	0.018263	1.210123	O/U
L-Lysine monohydrochloride	2.247586	0.034353	1.141402	B&C
17-Octadecynoic Acid	1.629519	0.025034	1.184805	O/U
N-Acetyl-D-lactosamine	1.569557	0.007832	1.324301	O/U

Note: The Capital letters represent the efficacy items of metabolites, which are as follows: A, Hemodynamic improvement; B, Efficient nutritional supplement; C, Hemostasis; D, Antibacterial;E, Anti-inflammatory; F, Short peptide-based regulation; G, Hepatoprotection; H, Hormone regulation; I, Neuromodulation, neuroprotection and tranquilization; J, Anti-tumor; K, Antioxidation; L, Antiviral. The 'O/U' was used to indicate other or unclear efficacies.

Tab. S4 The most up- and down-regulated metabolites between medicinal parts of Ang root (ARH vs. ARB) in the positive and negative ion mode and their efficacy annotations based on published literature and existing databases.

Metabolites	$\log_2$ FC	*P*	VIP	Efficacies
Up-regulated in ARB (positive ion mode)				
β-estradiol	17.314592	0.045989	1.310739	B&H
11-Deoxy-17-hydroxycorticosterone	15.664763	0.046827	1.370235	B&H
Erybraedin B	15.133892	0.024654	1.442352	J
Gly Ile Lys Arg	14.278889	0.013917	1.592609	B&F
1,2,4,5-Tetramethylbenzene	10.343807	0.000113	2.056331	O/U
Gly Asn Gly	9.492815	0.017614	1.604716	B&F

3-Thiatetradecanoic Acid	9.344582	0.036947	1.440843	O/U
Ser Val Lys Lys	8.143120	0.000665	1.987946	B&F
Ramipril	6.575434	0.000029	2.029121	A
PGD3	6.149392	0.020497	1.555526	O/U
Up-regulated in ARB (negative ion mode)				
(±)7-epi Jasmonic Acid	13.576486	0.030674	1.658267	O/U
Naringenin-7-O-Glucoside	13.442634	0.029282	1.556087	A&D&E&J&K
glutamyl-Asparagine	8.923301	0.030204	1.597819	B&F
Methyl Orsellinate	8.783322	0.046797	1.460511	D
Cyclic adenosine diphosphate ribose	8.615745	0.014263	1.691554	O/U
Alanyl-Tyrosine	8.067716	0.033856	1.457185	B&F
Lysyl-Proline	5.940672	0.001153	1.889881	B&F
Leucyl-Glutamate	4.977532	0.010633	1.744985	B&F
(+)-Citramailc acid	4.555716	0.017975	1.609874	O/U
S-Methyl-5'-thioadenosine	4.329962	0.031500	1.590671	B
Up-regulated in ARH (positive ion mode)				
7,8-Dihydroneopterin	6.752190	0.022732	1.490629	O/U
7-(4-Hydroxyphenyl)-1-phenyl-4-hepten-3-one	3.549869	0.001514	1.802306	D&J
Tryptophyl-Lysine	2.215550	0.020954	1.485630	B&F
Pinolenic Acid	2.142835	0.000921	1.801695	O/U
Histidinyl-Histidine	1.806271	0.048940	1.270510	B&F
Pro His Val Ile	1.772776	0.009542	1.606496	B&F
L-alpha-Glutamyl-L-tyrosine	1.745868	0.011273	1.579769	B&F
Phenylalanyl-Asparagine	1.533122	0.000226	1.898340	B&F
Flavanone	1.439437	0.031369	1.470842	A&D&E&K&J&L
Adenylsuccinic acid	1.408723	0.035745	1.382200	O/U
Up-regulated in ARH (negative ion mode)				
Manumycin A	4.692444	0.010880	1.804963	D&J

(±)Abscisic Acid	1.768652	0.026070	1.661566	O/U
Aloin A	1.491532	0.000937	2.018043	C
PGF1a	1.424410	0.018539	1.642573	A&H
Malic acid	1.194999	0.001120	2.036700	C
2-hydroxyhexadecanoic acid	0.984053	0.043788	1.321637	O/U
D-Glycerate 2-phosphate	0.914763	0.004375	1.846519	O/U
Fluoxetine	0.845488	0.011991	1.780673	I
Succinic acid	0.705385	0.018459	1.707086	C
L-Malic acid	0.310647	0.000020	2.177900	C

Note: The Capital letters represent the efficacy items of metabolites, which are as follows: A, Hemodynamic improvement; B, Efficient nutritional supplement; C, Hemostasis; D, Antibacterial; E, Anti-inflammatory; F, Short peptide-based regulation; G, Hepatoprotection; H, Hormone regulation; I, Neuromodulation, neuroprotection and tranquilization; J, Anti-tumor; K, Antioxidation; L, Antiviral. The 'O/U' was used to indicate other or unclear efficacies.

Tab. S5 Metabolites (positive ion mode) in ART with superior content over both ARH and ARB, and annotation of their efficacy.

Metabolites	ARH vs. ART		ARB vs. ART		Efficacies
	$\log_2$ FC	*P*	$\log_2$ FC	*P*	
Corchorusoside E	28.69	0.00110	20.76	0.00114	A
Ile Val Val Phe	28.42	0.00616	19.42	0.00898	B&F
6-pentadecyl Salicylic Acid	26.59	0.01157	19.11	0.01229	A&D
pos_7956 (potential structural analogues of corticosterone)	26.03	0.00035	18.89	0.00035	H
His His Arg Lys	27.95	0.00071	15.35	0.00055	B&F
pos_8029 (potential structural analogue of U-73122)	29.62	0.00001	12.60	0.00001	A&J
Amygdalin	17.82	0.02832	11.00	0.00115	A&E&J
O-Phospho-L-threonine	16.82	0.00012	9.51	0.00004	B

p-Cymene	13.03	0.04011	9.58	0.04366	O/U
5'-Prenyllicodione	11.71	0.00004	10.62	0.00002	O/U
Tyr His Tyr Gly	15.19	0.00635	6.58	0.01478	B&F
pos_7365 (potential structural analogue of sulfasalazine)	18.89	0.00146	2.43	0.00430	D&E
Cyclo(D-a-aspartyl-L-prolyl-D-valyl-L-leucyl-D-tryptophyl)	13.62	0.01091	4.20	0.01412	A
myriocin (from medicinal plant-microbial ecosystem)	10.81	0.03330	6.31	0.04253	A&D&E&L
4-Isopropylbenzyl alcohol	10.40	0.01018	6.44	0.01228	D&E
Trans-Zeatin	13.89	0.00561	1.71	0.01146	O/U
pos_6076 (structural analogue of atorvastatin)	12.24	0.00000	3.31	0.00000	A
Amlodipine	9.97	0.00074	5.32	0.02297	A
Glu His Ala Ser	5.29	0.00304	7.79	0.00331	B&F
Estriol 16.alpha.-(.beta.-D-glucuronide)	10.68	0.00893	2.01	0.01080	A&H
Sesaminol glucoside	2.11	0.02312	10.20	0.02406	D
Marmesin rhamnoside	7.55	0.00434	3.58	0.00479	A&K
1,3,5(10)-Estratrien-3,17.beta.-diol 17-glucosiduronate	6.30	0.00001	4.65	0.00005	H
Populin	8.18	0.00000	2.10	0.01052	O/U
Phenacetine	6.89	0.00240	3.17	0.00261	O/U
Gamma-Tocopherol	7.17	0.00004	2.73	0.00002	A
Methionyl-Methionine	7.83	0.00000	2.04	0.00486	K
Prenyl arabinosyl-(1->6)-glucoside	7.44	0.00005	2.35	0.00006	D
(Z)-1,5-Tridecadiene	2.03	0.02668	5.88	0.02341	D

17-beta-Estradiol-3-glucuronide	3.89	0.00006	3.56	0.00007	H
11beta-hydroxy-androst-4-ene-3,17-dione	4.76	0.00369	2.47	0.00786	H
Aristolindiquinone	4.46	0.00442	2.72	0.00744	O/U
15-deoxy-δ-12,14-PGJ2	4.16	0.01206	2.73	0.02138	O/U
8-8'-Dehydrodiferulic acid	3.91	0.00819	2.98	0.01056	A&E&J
Licoleafol	4.38	0.00006	2.37	0.00005	A&E&K
2,6-Dimethoxyquinone	3.68	0.00079	3.05	0.00090	A&D&E&J
Rhodinyl phenylacetate	3.89	0.01270	2.65	0.01894	H
Tobramycin	3.39	0.00147	2.99	0.00001	D
Ile His Met Thr	4.08	0.01408	2.13	0.01092	B&F
Isosafrole	3.63	0.00032	2.39	0.00153	O/U
2,3-dihydrobenzofuran	3.46	0.00187	2.18	0.00360	O/U
Anisomycin	3.71	0.00172	1.59	0.01186	D
Asp Asp His Ala	3.33	0.00081	1.80	0.00211	B&F
Asp His Gly Gly	3.19	0.00150	1.88	0.00299	B&F
Asparaginyl-Alanine	2.69	0.00697	2.28	0.00297	B&F
7-Ethoxy-4-methyl-2H-1-benzopyran-2-one	2.45	0.00006	2.20	0.00024	O/U
Valyl-Aspartate	2.76	0.00022	1.88	0.00025	B&F
Arachidonic Acid (peroxide free)	2.94	0.00000	1.68	0.00000	A&J
alpha-curcumene	3.39	0.00242	1.06	0.04074	A
Val Leu Leu Cys	2.46	0.00038	1.98	0.00069	B&F
4-Methylesculetin	2.71	0.00492	1.70	0.01154	E&K
Isoeugenyl acetate	2.88	0.00000	1.38	0.03127	O/U
Glu Asn Asp His	2.72	0.00076	1.45	0.00185	B&F

(2RS,5RS)-(E)-2-(2-Phenylethenyl)-1,3-dioxan-5-ol	2.80	0.00000	1.31	0.00473	E
2'-Deoxyuridine	2.12	0.02143	1.78	0.03224	O/U
Methylisoeugenol	2.51	0.00001	1.31	0.02216	O/U
18alpha.-Glycyr-rhetinic acid	2.26	0.00992	1.56	0.01867	D&E&J&K&L
3-O-Mycarosylery-thronolide B	2.15	0.00026	1.56	0.00000	D
p-Cresol	2.11	0.00191	1.56	0.00100	A
Eicosapentaenoic acid	2.56	0.00017	1.07	0.00090	A
Methyl-2-alpha-L-fu-copyranosyl-beta-D-galactoside	1.79	0.00502	1.74	0.00161	D&E&L
Cortisone acetate	1.93	0.00511	1.59	0.00751	E
Pentazocine	2.47	0.00142	1.05	0.02766	O/U
5(S),6(R),15(R)-Lipoxin A4	1.34	0.00742	2.12	0.00129	E
Cys Asn Met Tyr	1.88	0.00097	1.56	0.00185	B&F
(±)-Warfarin	1.67	0.00895	1.68	0.00001	A
Nystatin	1.57	0.01099	1.77	0.00864	D
Potassium sorbate	1.87	0.00003	1.39	0.00134	O/U
21-hydroxyallopreg-nanolone	1.78	0.01128	1.47	0.01716	H
6,10-Dimethyl-5(E),9-undecadien-2-one	1.43	0.01968	1.52	0.02054	O/U
β -Phenyl-gamma-Aminobutyric Acid	1.07	0.02953	1.64	0.03156	A&I
8E,10E-Tetradecadi-enal	1.32	0.02121	1.38	0.02187	O/U
2-Isopropenyl-4a,8-dimethyl-1,2,3,4,4a,5,6,7-oc-tahydronaphthalene	1.57	0.00227	1.10	0.00615	O/U
Caproic acid	1.18	0.03797	1.46	0.02849	O/U

MGDG(18:3(9Z,12Z,15Z)/18:3(9Z,12Z,15Z))	1.43	0.00660	1.12	0.01322	O/U
3-Phenylpropanoic acid	1.20	0.00198	1.26	0.00139	O/U
5,7-Diethyl-9-methyl-3E,5E,7E,9E-tridecatetraene	1.14	0.01774	1.22	0.01800	O/U
Farnesyl acetone	1.15	0.01416	1.20	0.01541	O/U
D-Pinitol	1.13	0.01566	1.22	0.02773	O/U

Note: The Capital letters represent the efficacy items of metabolites, which are as follows: A, Hemodynamic improvement; B, Efficient nutritional supplement; C, Hemostasis; D, Antibacterial; E, Anti-inflammatory; F, Short peptide-based regulation; G, Hepatoprotection; H, Hormone regulation; I, Neuromodulation, neuroprotection and tranquilization; J, Anti-tumor; K, Antioxidation; L, Antiviral. The 'O/U' was used to indicate other or unclear efficacies.

Tab. S6 Metabolites (negative ion mode) in ART with superior content over both ARH and ARB, and annotation of their efficacy.

Metabolites	ARH vs. ART		ARB vs. ART		Efficacies
	log_2 FC	*P*	log_2 FC	*P*	
Genkwanin	18.01	0.00535	20.77	0.00550	E
Levofloxacin	21.12	0.01069	14.11	0.01160	D
(±)7-epi Jasmonic Acid	24.06	0.01934	10.48	0.02147	O/U
Flavin mononucleotide (FMN)	15.44	0.02362	17.96	0.02380	O/U
2'-O-methylguanosine	16.48	0.00000	13.58	0.00000	O/U
Zidovudine	18.52	0.00033	10.75	0.00021	L
7-Methylguanosine	16.32	0.00016	12.58	0.00168	O/U
Bosentan	17.15	0.00034	9.31	0.00557	A
Lisinopril	16.84	0.00003	9.35	0.00005	A
Homocitrate	16.79	0.00000	8.83	0.00028	A
Sappanone A Dimethyl Ether	18.38	0.00000	6.72	0.00000	E&I&K
N2,N2-Dimethylguanosine	16.10	0.00005	8.79	0.00013	O/U
L-Anserine	13.06	0.02006	8.92	0.02370	A&I
Ile-Ala-Arg	14.82	0.00711	4.07	0.00860	B&F

Zingerone	12.89	0.00001	4.95	0.02636	A&D&G &I&K
Valeroyl Salicylate	8.34	0.01593	7.77	0.01046	O/U
Adenine	12.39	0.00003	3.53	0.00001	B
3-Methyluridine	11.67	0.00012	3.82	0.00016	O/U
Phenylalanyl-Histidine	10.51	0.01332	3.37	0.00400	B&F
cis-9-Palmitoleic acid	5.83	0.00615	6.14	0.00534	A&D&E
Deoxysappanone B 7,3'-Dimethyl Ether	9.22	0.01220	1.81	0.00248	A&E&I
9(S)-HOTrE	7.52	0.00560	2.97	0.00778	O/U
Isoleucyl-Lysine	3.05	0.00385	6.66	0.00335	B&F
Indole-3-acetamide	8.72	0.00825	0.71	0.01016	O/U
Asn-Trp-OH	4.88	0.00000	4.43	0.00000	B&F
S-Methyl-5'-thioadeno-sine	6.66	0.00130	2.33	0.00270	O/U
myo-Inositol	2.60	0.00163	5.32	0.00214	A
5-Methyltetrahydro-folate (5-Methyl-THF)	5.31	0.01889	2.34	0.00078	A&B&I&J &K
Glycyl-Tyrosine	4.08	0.00001	2.66	0.00002	B&F
Leucyl-Glutamate	5.79	0.00105	0.81	0.01828	B&F
9(S)-HpOTrE	3.61	0.01023	2.93	0.01375	A
Raffinose	3.84	0.00000	2.63	0.00000	O/U
TyrMe-Asp-OH	4.79	0.00007	1.68	0.00021	B&F
Scytalone	4.13	0.00145	2.07	0.00478	O/U
Butabarbital	3.15	0.01163	2.55	0.01015	I
Vidarabine	3.27	0.00004	2.40	0.00002	L
Tyrosyl-Serine	3.68	0.00019	1.82	0.00296	B&F
Lysyl-Serine	4.15	0.00003	1.28	0.00135	B&F
Acadesine (Drug)	3.87	0.01246	1.50	0.04438	A&E
(-)-Riboflavin	3.69	0.00302	1.52	0.01577	K
HoPhe-HoPhe-OH	2.67	0.00409	2.35	0.00487	B&F
Acetyl-L-tyrosine	2.52	0.00043	2.37	0.00228	O/U

D-Glucono-1,5-lactone	2.51	0.02802	2.14	0.04404	K
Glutathione	3.03	0.00123	1.58	0.00313	K
Uridine 5'-monophos-phate	2.71	0.00004	1.75	0.00011	O/U
Tyrosyl-Phenylalanine	2.79	0.00006	1.45	0.00026	A
Lawsone	2.61	0.00023	1.50	0.00617	D&K
Coumarin	2.41	0.00069	1.40	0.01057	E&J&K
Protionamide	2.08	0.00170	1.71	0.00316	D
Sinensetin	2.58	0.00558	1.06	0.00042	A&D&E&J&K&L
Oxoglutaric acid	2.47	0.00000	1.16	0.00001	B
PG(18_2(9Z,12Z)_18_0)	2.17	0.00334	1.43	0.00972	O/U
Phenylalanyl-Trypto-phan	2.42	0.00015	1.13	0.00016	B&F
Streptozocin	1.72	0.00119	1.67	0.00290	D
Salidroside	2.11	0.00438	1.07	0.03132	A&J
Glycerol 3-phosphate	1.48	0.02321	1.59	0.03384	O/U
Doxycycline	1.79	0.00152	1.11	0.00463	D
D-Neopterin	1.48	0.00110	1.37	0.00127	E&K
16b-Hydroxyestradiol	1.67	0.00011	1.11	0.01033	H
Ketoprofen	1.23	0.02104	1.10	0.01462	E
Naringin	1.13	0.00678	1.19	0.01057	A&J&K

Note: The Capital letters represent the efficacy items of metabolites, which are as follows: A, Hemodynamic improvement; B, Efficient nutritional supplement; C, Hemostasis; D, Antibacterial; E, Anti-inflammatory; F, Short peptide-based regulation; G, Hepatoprotection; H, Hormone regulation; I, Neuromodulation, neuroprotection and tranquilization; J, Anti-tumor; K, Antioxidation; L, Antiviral. The 'O/U' was used to indicate other or unclear efficacies.

Tab. S7 Metabolites (positive ion mode) in ARH and ARB with superior content over ART, and annotation of their efficacy.

Metabolites	ART vs. ARH		ART vs. ARB		Efficacies
	log_2 FC	*P*	log_2 FC	*P*	
Virginiamycin S1	24.39	0.01314	23.86	0.01913	D
L-alpha-Glutamyl-L-tyrosine	12.82	0.00170	11.08	0.01271	B&I
Tryptamine	7.21	0.00000	7.25	0.00540	C&I
Nalpha-Methylhistidine	6.84	0.01951	6.45	0.02354	K
DL-Arginine	4.57	0.00036	4.30	0.00339	C&B&G
Lys Ser Gly	4.84	0.00065	3.76	0.00935	B
Homatropine	3.60	0.00014	3.28	0.00216	O/U
L-Arginine	3.13	0.00023	3.09	0.00000	B&C&G
D-Lysine	3.52	0.00210	2.55	0.04702	B&C
N-Trimethyl-2-aminoethylphosphonate	2.84	0.00244	2.89	0.00449	G
Kynurenic acid	3.21	0.00004	2.45	0.00800	I
L-Isoleucine	3.16	0.00108	2.38	0.03433	B
Citrulline	2.67	0.00003	2.37	0.00430	B&G
L-Proline	2.30	0.00004	2.09	0.00127	B
1-Palmitoyl-sn-glycero-3-phosphocholine	1.95	0.02481	2.01	0.00245	G
Sepiapterin	2.50	0.00120	1.41	0.03252	C
Ser Glu His Thr	2.25	0.00003	1.32	0.03663	B&F
V-Pyrro/No	1.80	0.00000	1.60	0.00042	O/U
CDP-Ethanolamine	1.91	0.01827	1.47	0.01011	O/U
Trp Gly Phe	1.68	0.00018	1.67	0.04182	B&F
5-Methoxyindoleacetate	1.80	0.00195	1.55	0.01005	I

1-O-Feruloyl-β-D-glucose	1.91	0.01201	1.41	0.02015	E&K
11-Deoxycortisol	2.09	0.00604	1.12	0.02914	H
Citrinin	1.81	0.00147	1.36	0.03481	D&I&J
1-Linoleoylglycerophos-phocholine	1.78	0.00004	1.35	0.00833	B
LysoPE(18:2(9Z,12Z)/0:0)	1.80	0.00017	1.23	0.00796	O/U
Phytosphingosine	1.61	0.03593	1.15	0.00010	J
Benzyl acetate	1.66	0.00003	1.00	0.03051	O/U
PE(17:0/0:0)	1.48	0.01305	1.18	0.00199	O/U
3-Methyloxindole	1.29	0.00001	1.21	0.03420	O/U
LysoPE(15:0/0:0)	1.49	0.01420	1.01	0.00599	O/U
5-O-Methylleridol	1.39	0.02530	1.04	0.00928	O/U
Isoquinoline N-oxide	1.21	0.00000	1.08	0.02299	O/U
Tacrine	1.09	0.02060	1.10	0.00372	I

Note: The Capital letters represent the efficacy items of metabolites, which are as follows: A, Hemodynamic improvement; B, Efficient nutritional supplement; C, Hemostasis; D, Antibacterial; E, Anti-inflammatory; F, Short peptide-based regulation; G, Hepatoprotection; H, Hormone regulation; I, Neuromodulation, neuroprotection and tranquilization; J, Anti-tumor; K, Antioxidation; L, Antiviral. The 'O/U' was used to indicate other or unclear efficacies.

Tab. S8 Metabolites (negative ion mode) in ARH and ARB with superior content over ART, and annotation of their efficacy

Metabolites	ART vs. ARH		ART vs. ARB		Efficacies
	$\log_2$ FC	*P*	$\log_2$ FC	*P*	
L-Citrulline	17.06	0.02132	18.05	0.00825	B
D-Glycerate 2-phosphate	10.94	0.00005	10.03	0.00125	O/U
Salannin	11.12	0.04029	8.56	0.04372	D&J&L
(±)Abscisic Acid	7.92	0.00829	6.15	0.00842	O/U
L-Lysine monohydrochloride	2.88	0.00109	2.25	0.03435	B&C
Nicotinamide adenine dinucleotide (NAD)	2.72	0.00158	2.34	0.01826	B&C
2,5-Dihydroxybenzoic acid	2.02	0.04328	2.38	0.00680	O/U
17-Octadecynoic Acid	2.25	0.00026	1.63	0.02503	A
N-Acetyl-D-lactosamine	2.09	0.00114	1.57	0.00783	B
7,8-Dihydrofolate	1.88	0.00208	1.56	0.00111	A&B&I&K&J
Folinic acid	1.66	0.00542	1.52	0.01086	B&D&E&J&L
S-(1,2-Dicarboxyethyl)Glutathione	1.65	0.01148	1.51	0.00041	B&G&K
Tryptophyl-Glutamate	1.94	0.00706	1.13	0.04517	B&F
4-O-.beta.-Galactopyranosyl-D-mannopyranose	1.61	0.01616	1.29	0.00317	B
alpha-Mangostin	1.46	0.01660	1.40	0.00353	B&D&E&J&K&L
Pyridoxamine 5'-phosphate	1.40	0.00790	1.13	0.00910	B&K
L-Tyrosine	1.36	0.00114	1.07	0.02254	B
L-Tryptophan	1.11	0.00126	1.29	0.02417	B&I
PE(18_1(9Z)_0_0)	1.19	0.00065	1.18	0.00000	O/U
Indole	1.01	0.00029	1.07	0.03895	E

Note: The Capital letters represent the efficacy items of metabolites, which are as follows: A, Hemodynamic improvement; B, Efficient nutritional supplement; C, Hemostasis; D, Antibacterial; E, Anti-inflammatory; F, Short peptide-based regulation; G, Hepatoprotection; H, Hormone regulation; I, Neuromodulation, neuroprotection and tranquilization; J, Anti-tumor; K, Antioxidation; L, Antiviral. The 'O/U' indicated other or unclear efficacies. The 'O/U' was used to indicate other or unclear efficacies.

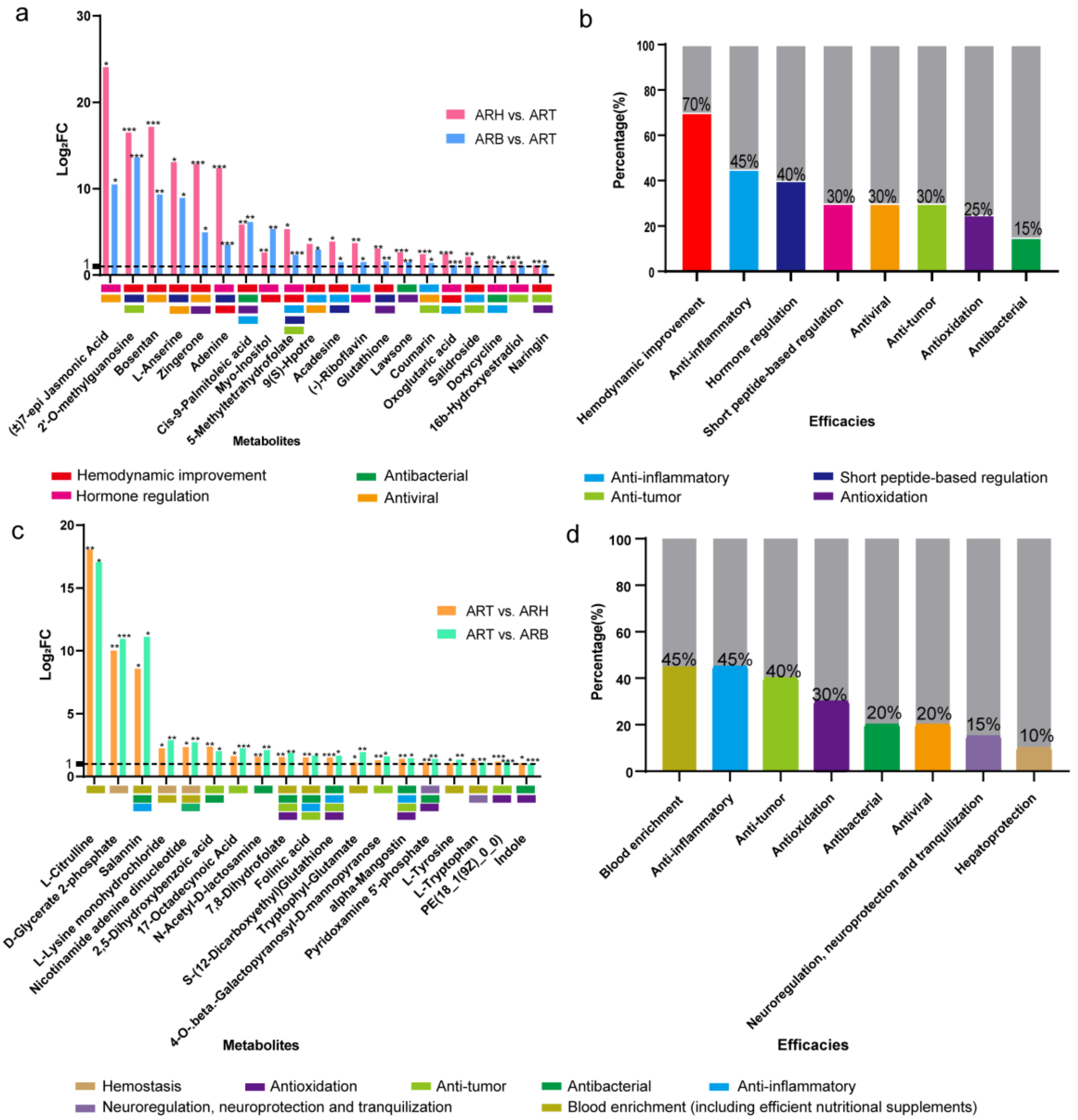

Fig. S4 Dominant metabolites (negative ion mode) and corresponding medicinal efficacy patterns in different medicinal parts of Ang root. a and b, The top metabolites in ART with higher contents than those in both ARH and ARB and an analysis of their medicinal efficacy patterns. c and d, Top metabolites in both ARH and ARB with higher contents than those in ART and an analysis of their medicinal efficacy patterns. The *, **, and *** denote p < 0.1, < 0.05 and < 0.01, respectively.

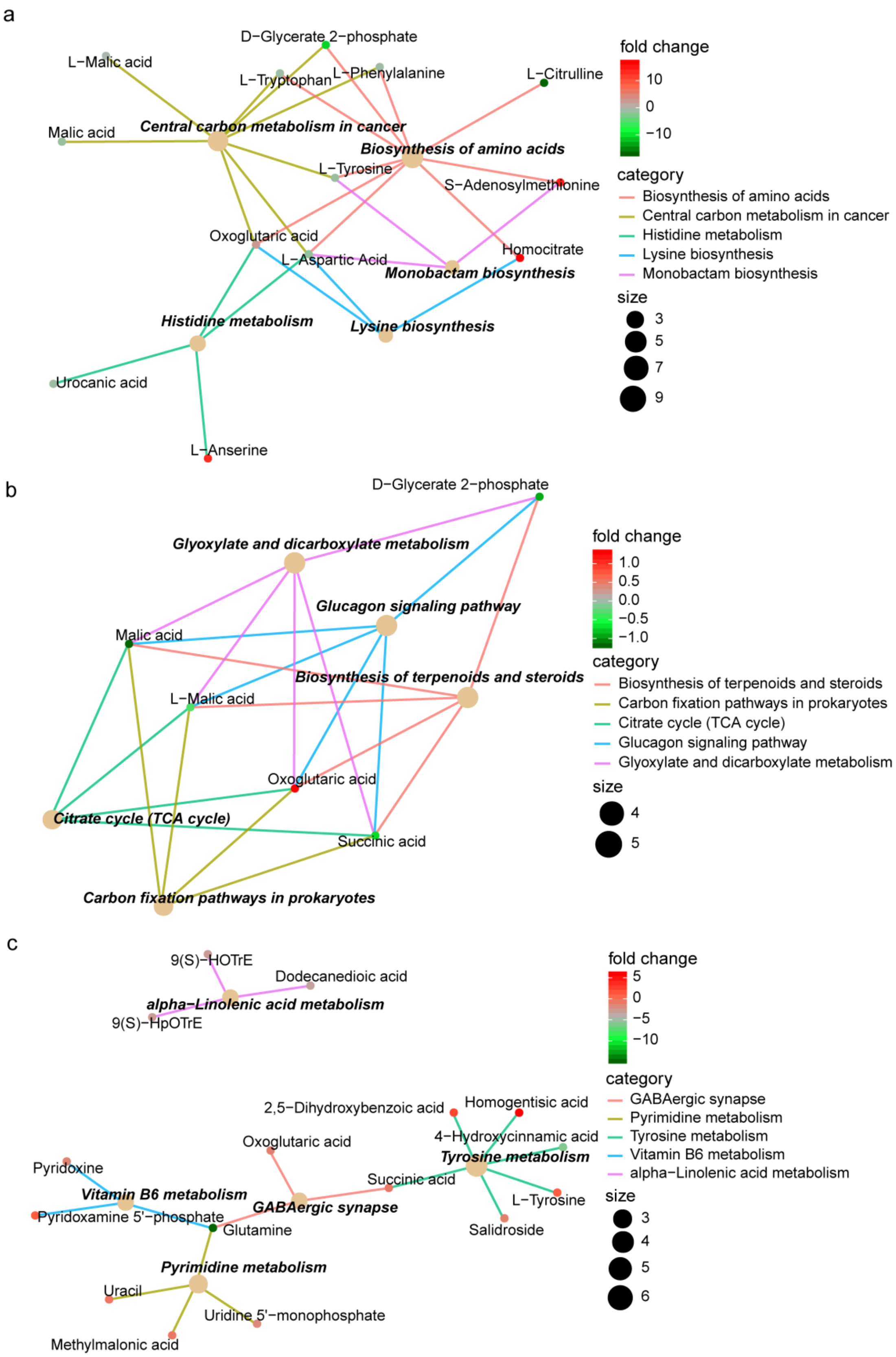

Fig. S5 KEGG enrichment analysis of the differential metabolites (negative ion mode) obtained from the different medicinal parts. a, b, and c correspond to ARH vs. ART, ARH vs. ARB, and ART vs. ARB, respectively.

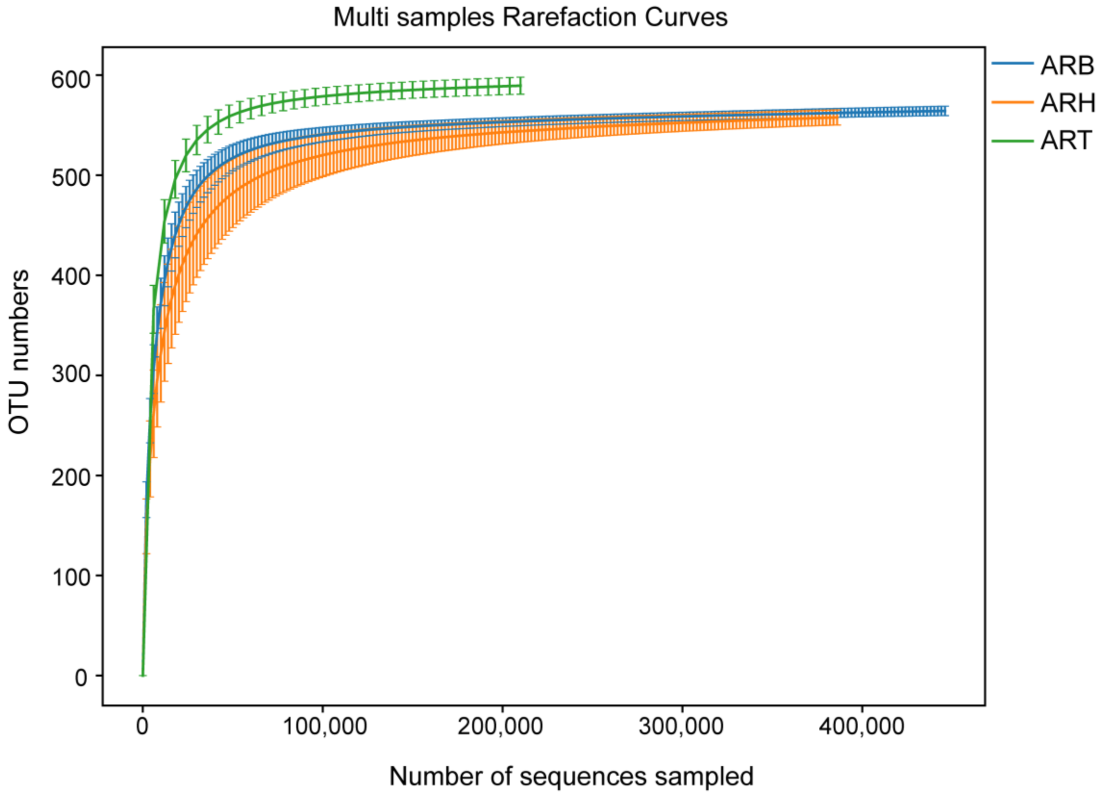

Fig.S6 Relationships between the ITS sequence numbers and the OTU numbers in the microbiome analyses for the different medicinal parts of Ang root. At the sequencing depth used in this study, the number of OTU tended to be flat, and the standard deviation was very small.

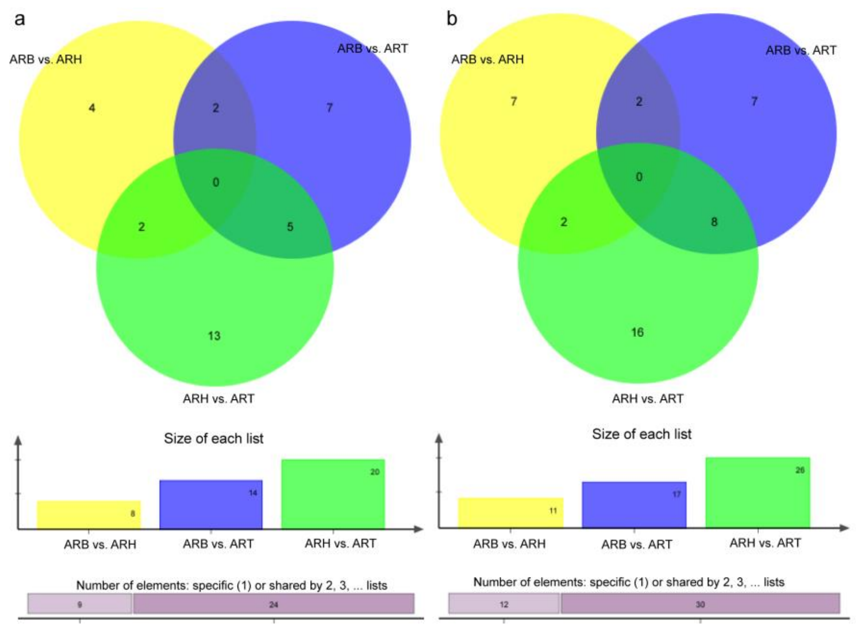

Fig. S7 Venn diagram showing the endophytic fungi at the genus (a) and species (b) levels in different medicinal

parts of Ang root.

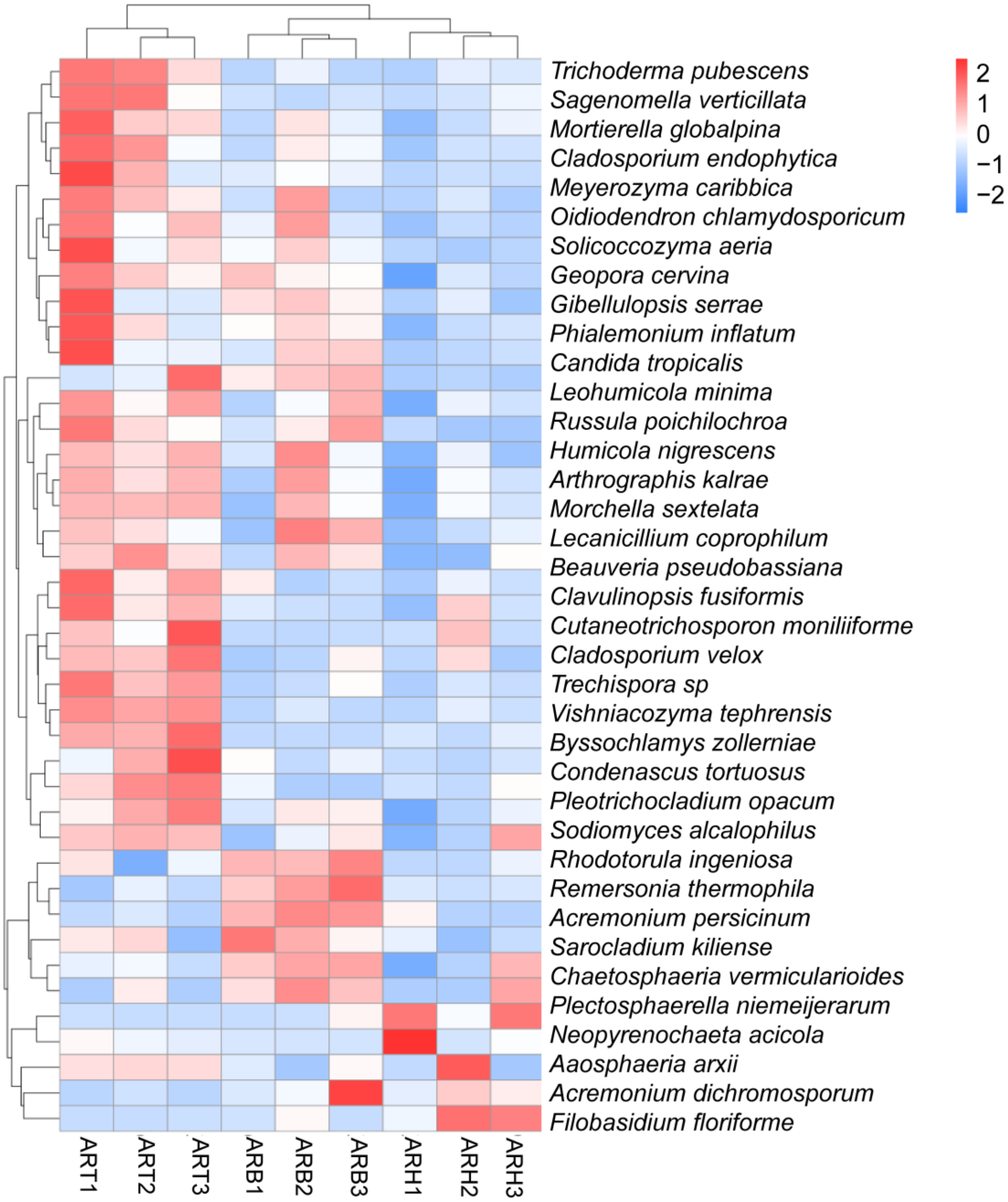

Fig. S8 Hierarchical cluster analysis of the samples from the different medicinal parts based on the abundance data for endophytic fungi at the species level.

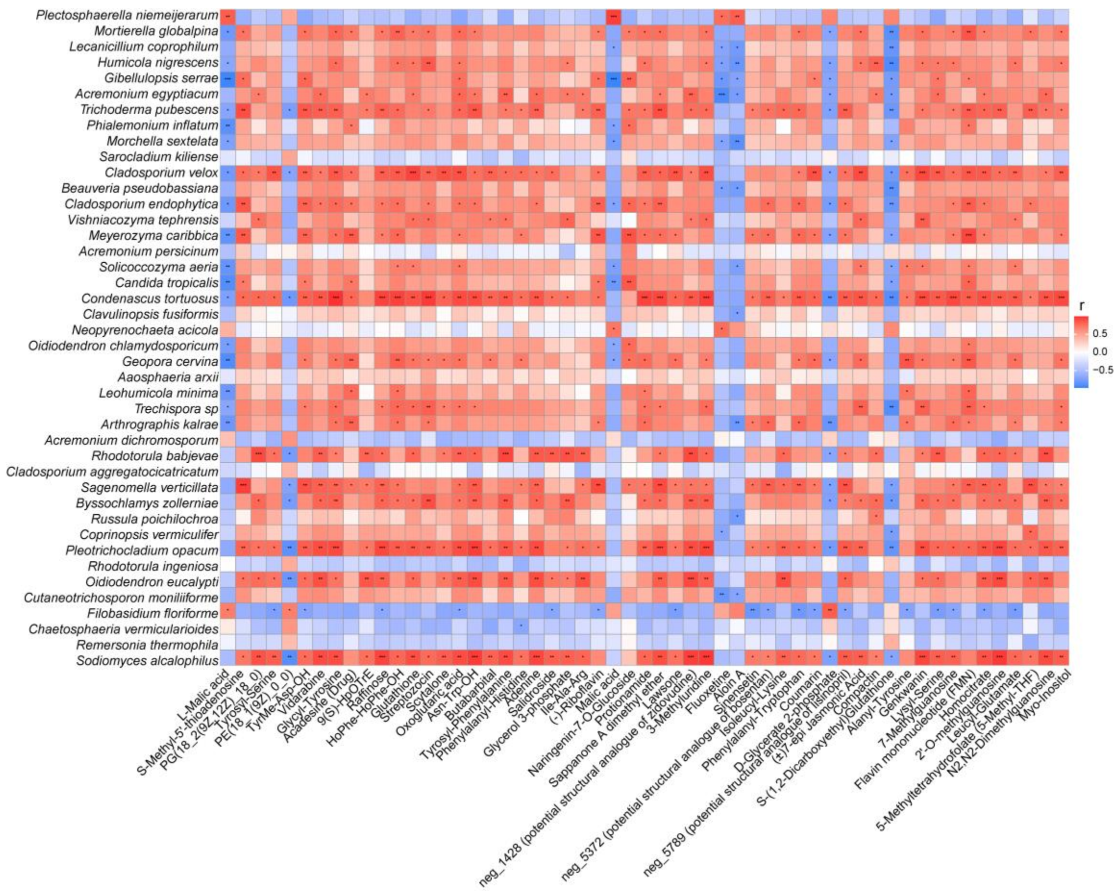

Fig. S9 Pearson correlation analysis of the differential metabolites (negative ion mode) and endophytic fungi (species level) based on variations among the different medicinal parts of Ang root. *, **, and *** represent significance levels of 0.1, 0.05, and 0.01, respectively.

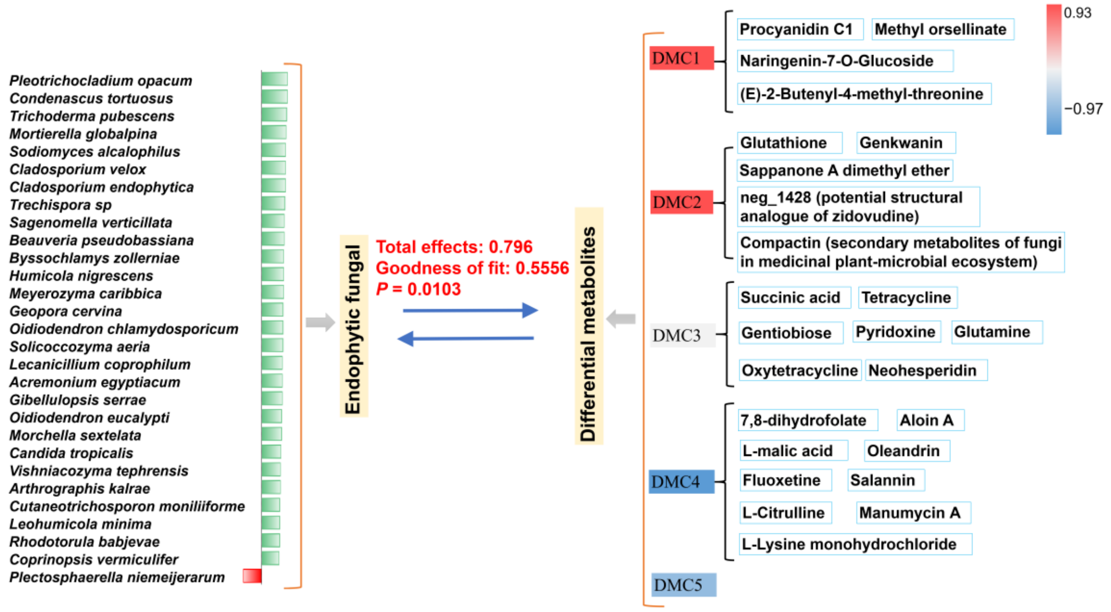

Fig. S10 Structural equation model for the endophytic fungi (species level) and metabolites (negative ion mode) based on the differences among the medicinal parts of Ang root. The green bar chart shows the indication values for the differential species to the microbial module; the red-blue colour gradation shows the indicator values for the differential metabolite clusters to the metabolite module.
